# Integrative network pharmacology and experimental verification to reveal the anti-inflammatory mechanism of ginsenoside Rh4

**DOI:** 10.3389/fphar.2022.953871

**Published:** 2022-08-31

**Authors:** Kwang-Il To, Zhen-Xing Zhu, Ya-Ni Wang, Gang-Ao Li, Yu-Meng Sun, Yang Li, Ying-Hua Jin

**Affiliations:** Key Laboratory for Molecular Enzymology and Engineering of the Ministry of Education, School of Life Sciences, Jilin University, Changchun, China

**Keywords:** network pharmacology, ginsenoside, inflammation, proinflamamtory cytokines, pharmacological mechanism

## Abstract

Inflammation is an innate immune response to infection, and it is the main factor causing bodily injury and other complications in the pathological process. Ginsenoside Rh4 (G-Rh4), a minor ginsenoside of Panax ginseng C. A. Meyer and Panax notoginseng, has excellent pharmacological properties. However, many of its major pharmacological mechanisms, including anti-inflammatory actions, remain unrevealed. In this study, network pharmacology and an experimental approach were employed to elucidate the drug target and pathways of G-Rh4 in treating inflammation. The potential targets of G-Rh4 were selected from the multi-source databases, and 58 overlapping gene symbols related to G-Rh4 and inflammation were obtained for generating a protein–protein interaction (PPI) network. Molecular docking revealed the high affinities between key proteins and G-Rh4. Gene ontology (GO) and pathway enrichment analyses were used to analyze the screened core targets and explore the target–pathway networks. It was found that the JAK-STAT signaling pathway, TNF signaling pathway, NF-κB signaling pathway, and PI3K-Akt signaling pathway may be the key and main pathways of G-Rh4 to treat inflammation. Additionally, the potential molecular mechanisms of G-Rh4 predicted from network pharmacology analysis were validated in RAW264.7 cells. RT-PCR, Western blot, and ELISA analysis indicated that G-Rh4 significantly inhibited the production of pro-inflammatory cytokines such as TNF-α, IL-6, and IL-1β, as well as inflammation-related enzymes in lipopolysaccharide (LPS)-stimulated RAW264.7 cells. Moreover, *in vitro* experiments evaluated that Ginsenoside Rh4 exerts anti-inflammatory effects *via* the NF-κB and STAT3 signaling pathways. It is believed that our study will provide the basic scientific evidence that G-Rh4 has potential anti-inflammatory effects for further clinical studies.

## Graphical Abstract

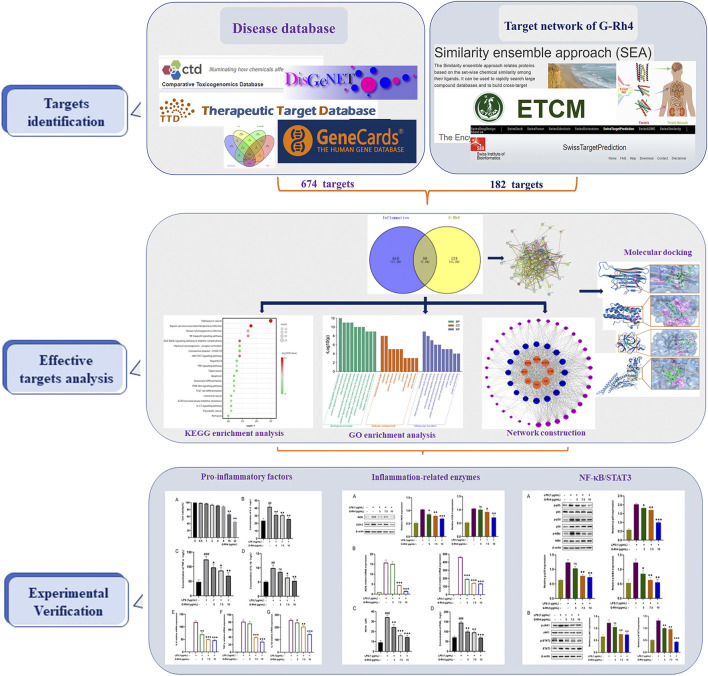

## Introduction

The occurrence of self-limited inflammation is critical for survival during physical injury and infection; however, the persistence of inflammation leads to several pivotal diseases that collectively represent the leading causes of disability and mortality worldwide (Furman et al., 2019; Panigrahy et al., 2020). Recent research has revealed that chronic inflammatory diseases have been recognized as the most important cause of death in the world today (Libby, 2021), and more than 50% of deaths are attributed to inflammation-related diseases such as ischemic heart disease, cancer, non-alcoholic fatty liver disease (NAFLD), and diabetes mellitus (GBD 2017 Causes of Death Collaborators, 2018). In addition, it was reported that typical biomarkers of acute inflammation can predict the incidence rate and mortality of various diseases (Arai et al., 2015), but this approach also has limitations. To address the limitations of assessment with a few selected inflammatory biomarkers, researchers have adopted a multi-dimensional approach, including analyzing a large number of inflammatory markers and then combining these markers into more reliable indicators representing high inflammatory activity (Morrisette-Thomas et al., 2014). The anti-inflammatory drugs, including non-steroidal anti-inflammatory drugs, have been developed and applied in clinics, but studies have shown that these drugs frequently cause adverse effects such as liver injury (Schmeltzer et al., 2016). Looking for more effective and safe anti-inflammatory drugs is an urgent issue in the global medical community.

Ginsenoside is the main active ingredient of ginseng, which displayed a broad spectrum of activities, such as cancer cell toxicity, anti-inflammation, and enhancing immunity. G-Rh4 is a trace saponin in white ginseng, and it became the most abundant triol-type ginsenoside after the heating process. Compared with other saponins, G-Rh4 has relatively better water solubility (Wang et al., 2022), suggesting its potential clinical applications. However, due to its low content in fresh or white ginseng and consequent preparation difficulty, there are few studies on the pharmacological effects of G-Rh4; especially, its anti-inflammatory activity and underlying mechanism are largely unknown.

Network pharmacology is the construction and analysis of biological networks based on network database retrieval and computer simulation calculation to study the mechanism of drug action. In particular, in determining the pharmacological mechanism and safety of Chinese medicine, the application of network analysis is a new paradigm for traditional Chinese medicine, from empirical medicine to evidence-based medicine (Wang et al., 2021). Therefore, network pharmacology research, as a new interdisciplinary approach, is of great value to the research and development of modern Chinese medicine (Sun et al., 2020; Wang et al., 2021). In this study, we clarified the drug target and multiple pathways of G-Rh4 in the treatment of inflammation by network pharmacology and the experimental approach.

## Methods

### Prediction of G-Rh4-related targets

**FIGURE 1:**
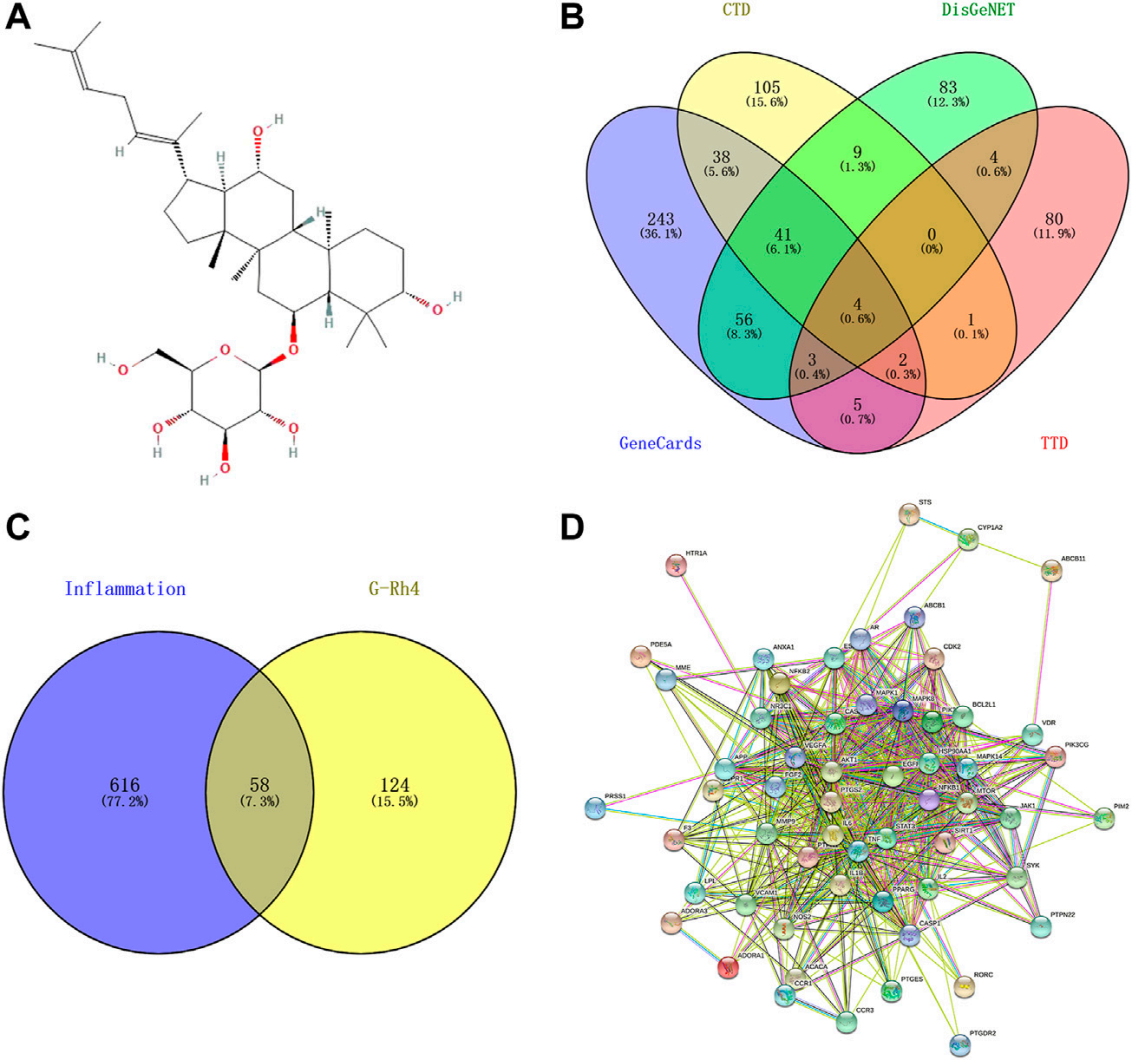
Targets screening involved in G-Rh4 for treating inflammation. **(A)** Molecule structure of G-Rh4. **(B)**Venn diagram of the potential inflammation-specific targets. **(C)** Fifty-eight overlapping target proteins between inflammation-related proteins and targets of G-Rh4. **(D)** Protein–protein networks of overlapping 58 target proteins. Edges: Interactions between protein(s) and protein(s).

The PubChem database (https://pubchem.ncbi.nlm.nih.gov/) was searched to obtain the two-dimensional structure diagram in SDF format and the canonical smiles of G-Rh4 (Figure 1A). The main potential targets of ginsenoside-Rh4 were identified using the Swiss Target Prediction (http://swisstargetprediction.ch/), the TargetNet (http://targetnet.scbdd.com/), the Similarity Ensemble Approach (SEA) (https://sea.bkslab.org/), and the Encyclopedia of Traditional Chinese Medicine (ETCM) (http://www.tcmip.cn/ETCM/index.php/Home/Index/) database.

### Acquisition of gene targets for inflammation

The genes related to inflammation were selected from the Therapeutic Target Database (TTD, http://db.idrblab.net/ttd/) (Wang Y. et al., 2019), the GeneCards (https://www.genecards.org/) (Rappaport et al., 2017), DisGeNET (https://www.disgenet.org/search) (Piñero et al., 2019), and the Comparative Toxicogenomics Database (CTD, http://ctdbase.org/search/) (Davis et al., 2021), where the database was searched using “inflammation” and “inflammatory” as the keywords.

### Screening of potential therapeutic targets

The target genes of G-Rh4 and the target information of inflammation were uploaded to Venny 2.1 (https://bioinfogp.cnb.csic.es/tools/venny/index.html) to obtain the target information of G-Rh4 intersecting with inflammation, and it is considered as the potential target of Rh4 in the treatment of inflammation.

### Network diagram of drug–disease PPI

The protein–protein interaction (PPI) network diagram was obtained through the STRING database (https://string-db.org/) (Szklarczyk et al., 2021). The TSV-format file was downloaded from the STRING database and imported into Cytoscape 3.8.0 for core target screening.

### Gene Ontology and KEGG enrichment analysis

The candidate targets were identified by using the DAVID v6.8 (https://david.ncifcrf.gov/) (Huang et al., 2009) to conduct GO and Kyoto Encyclopedia of Genes and Genomes (KEGG) enrichment analysis on the target of G-Rh4 in the treatment of inflammation. A threshold of *p* < 0.05 was used to identify key GO and KEGG pathways; GraphPad Prism 8 0.1 software was used to visualize the analysis results.

### Diagram of core drug targets

Using the software Cytoscape 3 8.0, the relationship network diagram of core drug targets was constructed and topological analysis was performed, after which the importance of targets was analyzed according to the degree value (Rahimmanesh and Fatehi, 2020).

### Molecular docking verification

First, PDB format of the key target proteins and MOL2 format of Ginsenoside Rh4 were obtained through the RCSR protein database (https://www.rcsb.org/) and the NCBI PubChem Compound database, and PyMOL software was employed to remove the water and ligands. After that, molecular docking was conducted between the treated proteins and active components by using AutoDock Vina software, and the binding energy was evaluated, where the value of <0 indicated that the receptors and compounds can bind by themselves (Liang et al., 2021), while the value of <–5.0 kcal/mol meant that they had good binding activities. The lower the binding energy, the greater the probability of binding and the more credible the result.

### Cell lines and culture

RAW264.7 cells were purchased from the Chinese Academy of Sciences Stem Cell Bank. DMEM containing 10% FBS and double antibodies (penicillin 100 U/mL and streptomycin 100 μg/ml) was used as a culture medium, and cells were cultured in an incubator at 37°C and 5% CO_2_.

### Assay to measure cell viability

The logarithmic period RAW264.7 cells (1 × 104 cells/well) were inoculated on 96-well plates and cultured for 24 h. After treatment with different concentrations of G-Rh4 in serum-free DMEM for 24 h, 20 μL of MTT (5 mg/ml; Sigma, USA) solution was added. After incubation for another 4 h, the culture medium was discarded and 150 μL DMSO (Sigma, USA) was added to each well. The absorbance at 550 nm was measured using a TECAN microplate reader (Maennedorf, Switzerland).

### Real-time quantitative polymerase chain Reaction

RAW264.7cells (2 × 105 cells/mL) were inoculated in 100-mm dishes and cultured for 24 h. LPS (1 μg/ml) was pretreated for 4 h and further treated with different concentrations (5, 7.5, and 10 μg/ml) of G-Rh4 for another 16 h. At the end of treatments, the total RNA of RAW264.7 cells was isolated with TRIzol (Invitrogen, Grand Island, NY, USA), and 5 µg total RNA was proceeded for cDNA synthesis with a High Capacity cDNA Reverse Transcription Kit (4368814, Applied Biosystems, Foster City, CA, USA). Real-time quantitative experiments were conducted according to the instructions of the 7,500 Real-time PCR system (Applied Biosystems, Foster City, CA, USA) to determine the mRNA expression. The primer sequences are shown in Supplementary Table S1.

### Determination of levels of IL-6, TNF-α, IL-1β, and PGE_2_ by ELISAs

RAW264.7 cells (2.5 × 105 cells/mL) were inoculated in 12-well plates and cultured for 24 h. LPS (1 μg/ml) was pretreated for 4 h and different concentrations of G-Rh4 were treated for 16 h. The levels of IL-6, TNF-α, IL-1β, and PGE2 in the supernatant were measured, respectively, according to the operation instructions of the ELISA kit (CLOUD-CLONE CORP., Wuhan, Hubei).

### Nitrite assay

The logarithmic period RAW264.7 cells (1 × 104 cells/well) were inoculated on 96-well plates and cultured for 24 h. The cells were pretreated with LPS (1 μg/ml) for 4 h and then treated with different concentrations of G-Rh4 for 16 h. Nitrite levels in the cell culture supernatants were measured using Griess assay. Subsequently, 50 μL of the culture medium were mixed with 50 μL reagents of Griess A and Griess B, followed by incubation for 10 min at room temperature (light protected). A wavelength of 540 nm was selected to detect the absorbance values using a microplate reader, and nitrite levels were measured using a standard curve prepared from sodium nitrite (Park et al., 2005).

### Western blot analysis

RAW264.7cells (7×10^5^ cells/well) were seeded in 6-well plates and cultured for 24 h. The cells were pretreated with LPS (1 μg/ml) for 4 h and treated with different concentrations of G-Rh4 for 16 h. At the end of treatments, the cells were collected in a 1.5-ml centrifuge tube and then centrifuged at 10,000 r/min (rpm) for 5 min at 4°C, and the supernatant was discarded. The supernatant was washed with PBS and centrifuged at 10,000 rpm for 5 min, after which the cells were lysed with the RIPA cell lysate supplemented with 1% PMSF for 50 min and centrifuged at 12,000 rpm for 15 min to collect the supernatant. The protein concentrations were detected using BSA (bovine serum albumin, Sigma) as a standard. Equal amounts of protein (30 µg) were taken and subjected to SDS-PAGE electrophoresis. Then, the protein was transferred to the PVDF membrane and sealed with 5% (w/v) skim milk in Tris-buffered saline containing 0.1% Tween 20 (TBST). The primary antibody was incubated overnight at 4°C. After washing with TBST buffer, the membranes were incubated with the secondary antibody at room temperature for 1 h. The protein bands were quantitatively analyzed by ECL chromogenic exposure in a dark chamber.

### Statistical analysis

The experimental data were obtained from independent triple-replicated experiments and were expressed as the mean ± SD. Statistical analyses were processed using GraphPad Prism 8.0, and the Student’s *t*-test statistical analysis method was used to compare groups. *p* < 0.05 showed that the difference was statistically significant.

## Results

### Prediction of G-Rh4 potential target

In this experiment, network pharmacology and an experimental approach were employed to elucidate the drug target and pathways of G-Rh4 against inflammation (Figure 1 **)**. A total of 182 potential targets of ginsenoside Rh4 (G-Rh4) were collected from the databases such as Swiss Target Prediction, TargetNet, SEA, and ETCM, and 674 inflammation-related targets were screened through databases including GeneCards, DisGeNET, Therapeutic Target Database (TTD), and Comparative Toxicogenomics Database (CTD) (Figure 1B). The potential targets of G-Rh4 were intersected with inflammation-related genes, and as a result, 58 intersected genes were obtained. The Venn diagram is drawn in Figure 1C, and the names of intersection genes are listed in Supplementary Table S2.

### Identification of G-Rh4 core targets against inflammation

**FIGURE 2:**
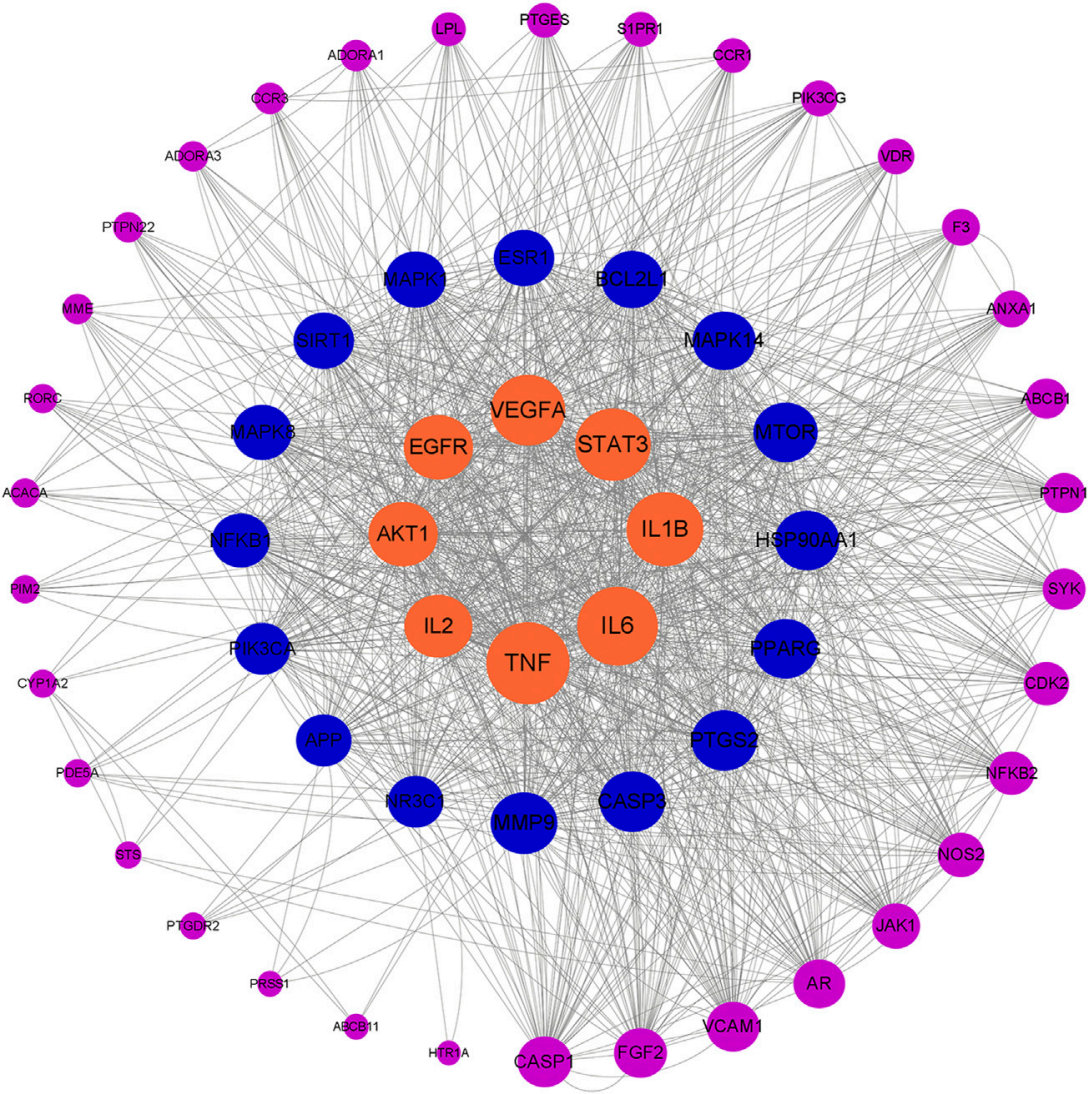
Network topology analysis of G-Rh4 treating inflammation; the target genes are sorted according to degree.

A total of 58 intersected target genes of G-Rh4 against inflammation were uploaded to the String database. Organization was set as *Homo sapiens*, and obtained the key targets (Figure 1D). The PPI data were imported into Cytoscape software, and the target node degree is taken as an important parameter of topology analysis to screen out the key nodes in the network, which are shown in Figure 2, where the size of the ellipse represents the major degree of the target genes.

### Gene Ontology and KEGG pathway enrichment analysis of the target genes of G-Rh4 against inflammation

**FIGURE 3:**
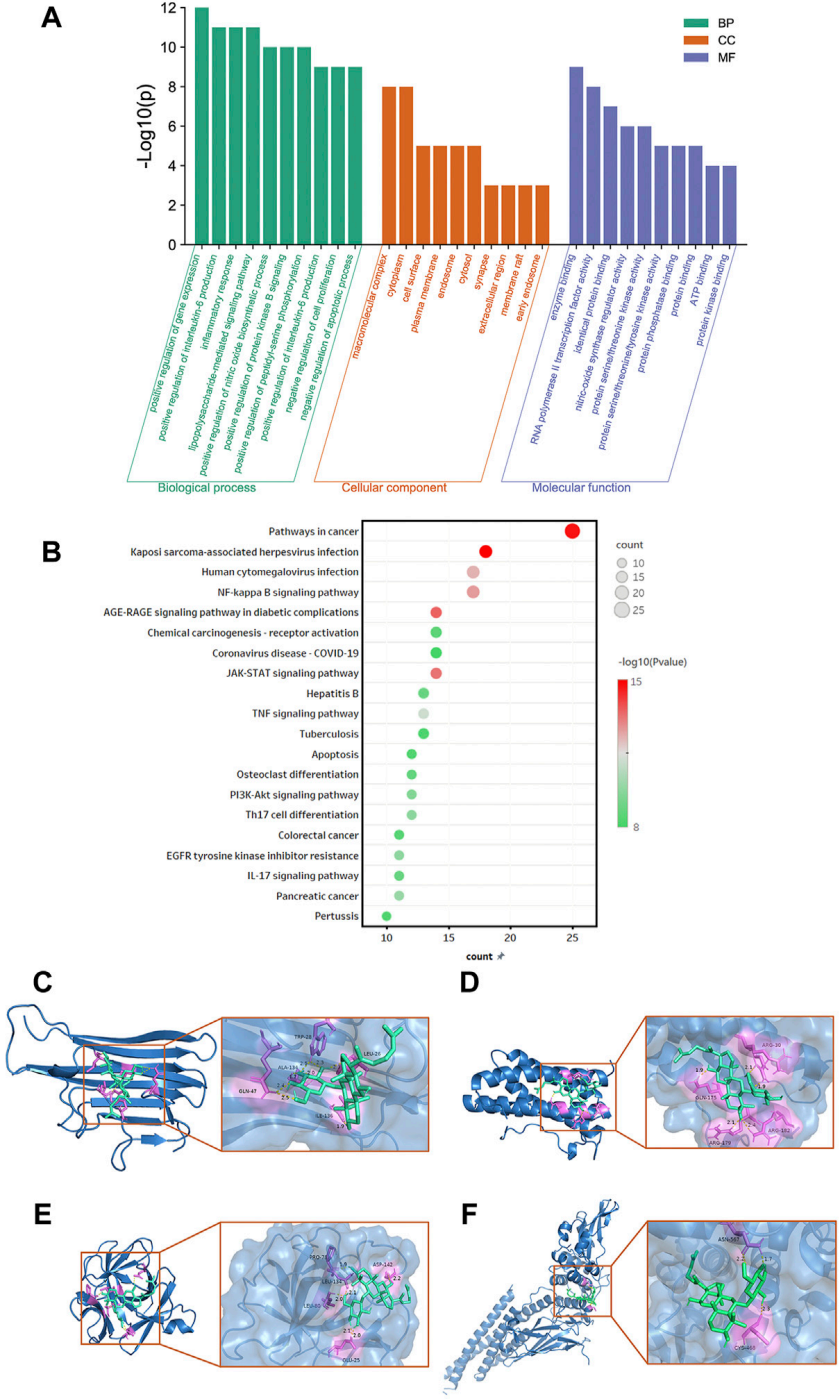
Functional enrichment analysis and docking simulation. Gene Ontology**(A)** and KEGG pathway enrichment **(B)** analysis of the target genes of G-Rh4 against inflammation. **(C–F)** The docking mode of G-Rh4 binding with target proteins; **(C)** TNF-α, **(D)** IL-6, **(E)** IL-1β, and **(F)** STAT3.

To further understand the biological processes involved in the screened key candidate targets above and their correlation with “inflammation,” we conducted analyses of GO and KEGG signaling pathways. The DAVID database was used to classify and count the GO function of 58 intersected genes of G-Rh4 in the treatment of inflammation (Supplementary Table S3). The pathways were visually analyzed to understand the functional distribution characteristics of different genes. The histogram for the pathway enrichment analysis was shown in Figure 3A. The GO analysis results indicated that the biological process (BP) terms were mainly associated with the positive regulation of the nitric oxide biosynthetic process, inflammatory response, positive regulation of interleukin-6 production, the lipopolysaccharide-mediated signaling pathway, and negative regulation of cell proliferation; the main cellular component (CC) terms were the macromolecular complex, cytoplasm, cell surface, plasma membrane, and endosome. Their primary molecular function (MF) was concentrated on enzyme binding, RNA polymerase II transcription factor activity, identical protein binding, nitric-oxide synthase regulator activity, and protein serine/threonine kinase activity.

In addition, to clarify the underlying involved pathway of inflammation targets treated by G-Rh4, the pathway enrichment analysis on target genes was conducted through the KEGG public database (Supplementary Table S4). A total of 58 intersected genes were imported into the DAVID database for KEGG pathway annotation analysis, after which the top 20 pathways were visually analyzed to obtain the pathway enrichment analysis bubble diagram (Figure 3B). It could be seen that common targets were mainly concentrated in the JAK-STAT signaling pathway, TNF signaling pathway, NF-κB signaling pathway, and PI3K-Akt signaling pathway.

### Molecular docking simulation

According to hub gene analysis, TNF-α, IL-6, IL-1β, and STAT3 are the key targets of G-Rh4 in the treatment of inflammation. Molecular docking simulation was applied to predict the binding ability between G-Rh4 and hub targets. The molecular docking of G-Rh4 with key targets was performed using AutoDock Vina software, and the binding energy was evaluated. The binding energy between G-Rh4 and TNF-α and IL-6 was –6.79 and –7.25 kcal/mol, respectively. The binding energy between G-Rh4 and IL-1β and STAT3 was –7.99 and –5.15 kcal/mol, respectively, suggesting that they have good binding activities (Table 1). The docking results were visualized using PyMOL software, as shown in Figures 3C–F.

**TABLE 1:** Result of molecular docking.

Targets	PDB	Binding energy (kcal/mol)
TNF-α	5M2J	−6.79
IL-6	1ALU	−7.25
IL-1β	2I1B	−7.99
STAT3	6NJS	−5.15

### Effect of G-Rh4 on the viability of RAW264.7 cells

**FIGURE 4:**
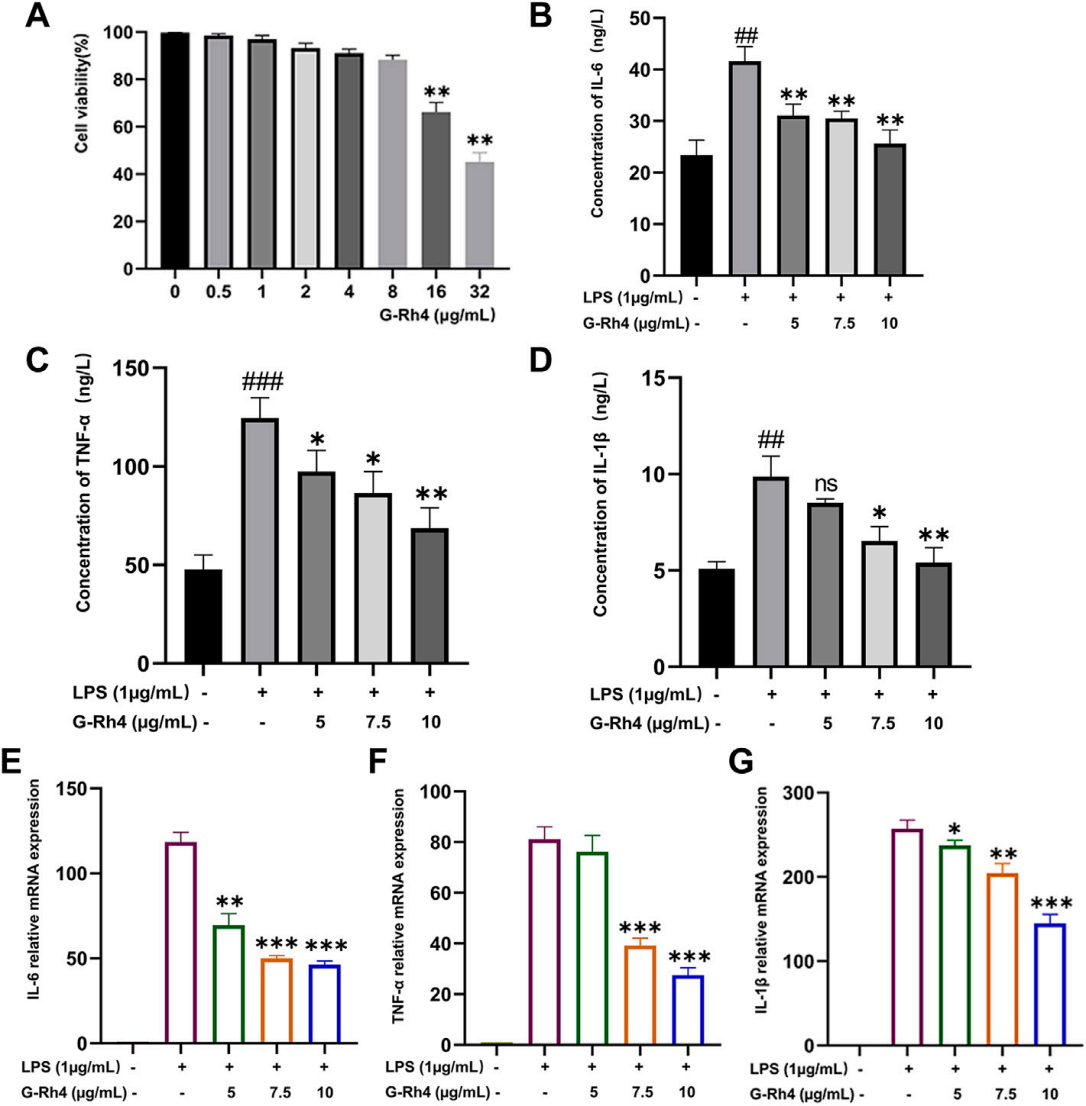
Effect of G-Rh4 on RAW264.7 cells. **(A)** Effect of G-Rh4 on cell viability. Cells were treated with different concentrations of G-Rh4 for 24 h. **(B–D)** Production of IL-6, TNF-α, and IL-1β in LPS-stimulated RAW264.7 cells was determined by ELISAs. **(E–G)** Effect of G-Rh4 on TNF-α, IL-6, and IL-1β mRNA levels in RAW264.7 cells. Data were presented as the mean ± SD values performed in triplicate (^##^ *p* < 0.01 and ^###^ *p* < 0.001 vs. control group, *p < 0.05, **p < 0.01, ***p < 0.001 vs. LPS group).

To study the effect of G-Rh4 on the viability of RAW264.7 macrophages, cells were treated with different concentrations of G-Rh4 for 24 h, and the cell activity was detected by MTT assay. As shown in Figure 4A, compared with the control group, G-Rh4 treatment showed no significant effect on the viability of macrophages at the concentration of 0.5–8 μg/ml (*p* > 0.05), indicating that G-Rh4 has no toxicity to RAW264.7 cells at this concentration.

### The production of IL-6, TNF-α, and IL-1β was inhibited by G-Rh4 treatment

LPS is a component of the cell wall of gram-negative bacteria, which can stimulate the activation of immune cells such as macrophages and cause a systemic inflammatory response. To study the effect of G-Rh4 on major inflammatory factors, the protein and mRNA levels of IL-6, TNF-α, and IL-1β in LPS-stimulated RAW264.7 cells were evaluated by ELISAs and RT-PCR. The results are shown in Figures 4B–D. The contents of TNF–α, IL-6, and IL-1β in LPS-stimulated RAW264.7 were significantly higher than those in the normal group (*p* < 0.01, *p* < 0.001), but after G-Rh4 treatment, they decreased significantly in a concentration-dependent manner (*p* < 0.05, *p* < 0.01). Also, the increased mRNA expression of the main target factor after stimulation of LPS decreased significantly with the addition of G-Rh4 (Figures 4E–G), which seems to support the experimental results of ELISAs.

### Effect of G-Rh4 on expression of iNOS/COX-2 and NO/PGE_2_ production in RAW264.7 cells

**FIGURE 5:**
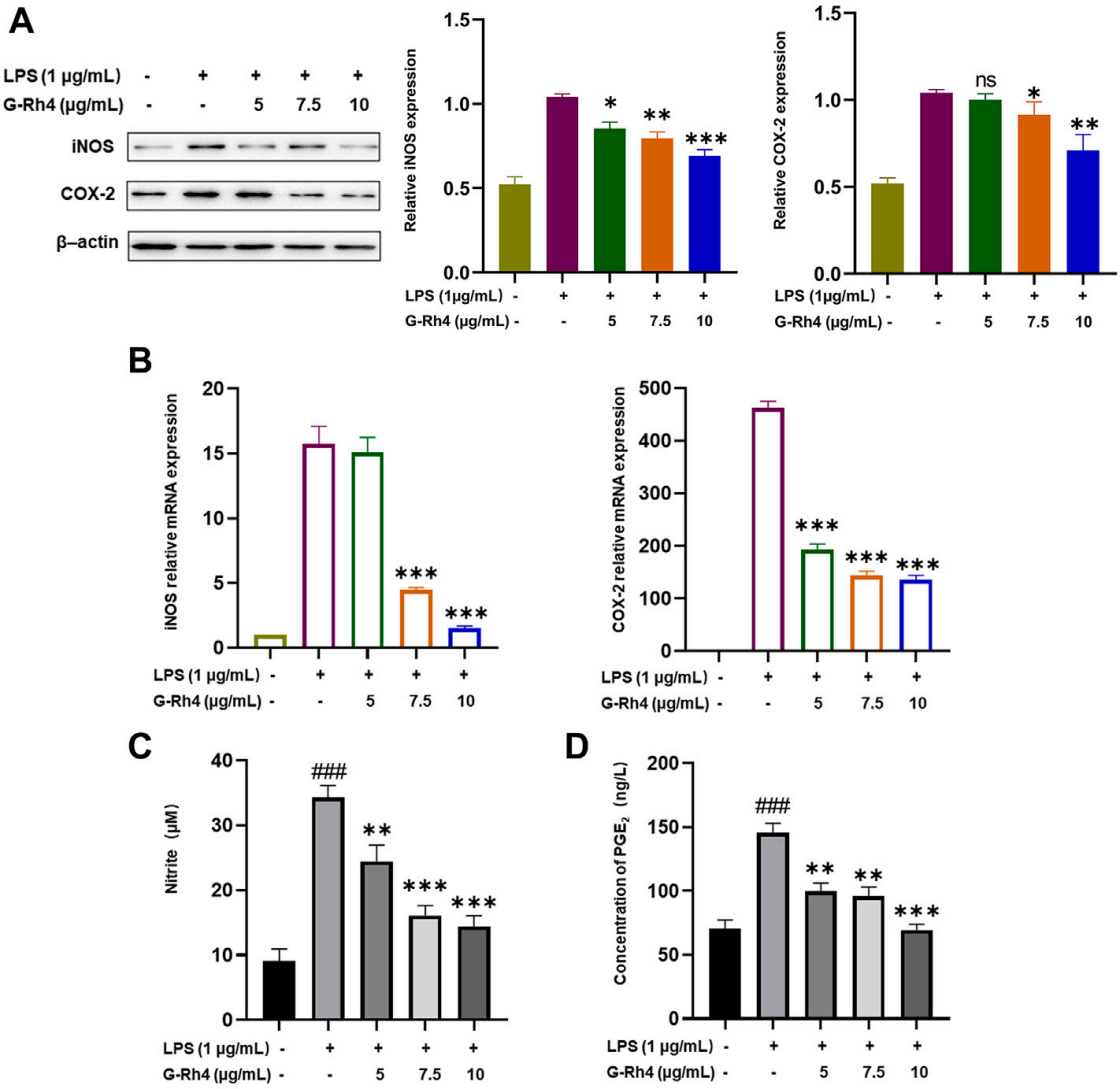
Inhibitory effects of G-Rh4 on NO/PGE_2_ production and iNOS/COX-2 expression in LPS-stimulated RAW264.7 cells. **(A)** Effect of G-Rh4 on iNOS and COX-2 protein levels in RAW264.7 cells. **(B)** Determination of iNOS and COX-2 mRNA levels in LPS-stimulated RAW264.7 cells. **(C)** NO levels in the culture medium evaluated by Griess reagent. **(D)** Effect of G-Rh4 on the release of PGE_2_ in RAW264.7 cells. Data were presented as the mean ± SD values performed in triplicate (^##^ *p* < 0.01 and ^###^ *p* < 0.001 vs. control group, *p < 0.05, **p < 0.01, ***p < 0.001 vs. LPS group).

The expression levels of iNOS and COX-2 in RAW264.7 cells were detected by Western blot and RT-PCR analysis. Compared with the LPS treatment group, a 5 μg/ml dose of G-Rh4 had no significant difference in inhibiting the expression of COX-2. But it decreased significantly at a dose of 7.5 μg/ml and iNOS levels were markedly reduced from those at the 5 μg/ml dose of G-Rh4 (Figure 5A). The mRNA expressions of iNOS and COX-2 were also decreased significantly at the 7.5 μg/ml dose of G-Rh4 compared with those in the LPS treatment group (*p* < 0.001) (Figure 5B). These results showed that G-Rh4 meaningfully inhibited the expression of inflammatory promoting enzymes in the inflammatory response. iNOS and COX-2 are the key enzymes involved in the synthesis of NO and PGE_2_, which are also the key factors causing inflammatory responses and individual pathological status. We investigated whether G-Rh4 regulates NO and PGE_2_ synthesis in LPS-stimulated RAW264.7 cells. LPS stimulation increased the production of NO and PGE_2_, and the release of NO and PGE_2_ was significantly reduced in a dose-dependent manner upon G-Rh4 treatment (Figures 5C,D).

### G-Rh4 inhibited the LPS-induced NF-κB and STAT3 activation in RAW264.7 macrophages

**FIGURE 6:**
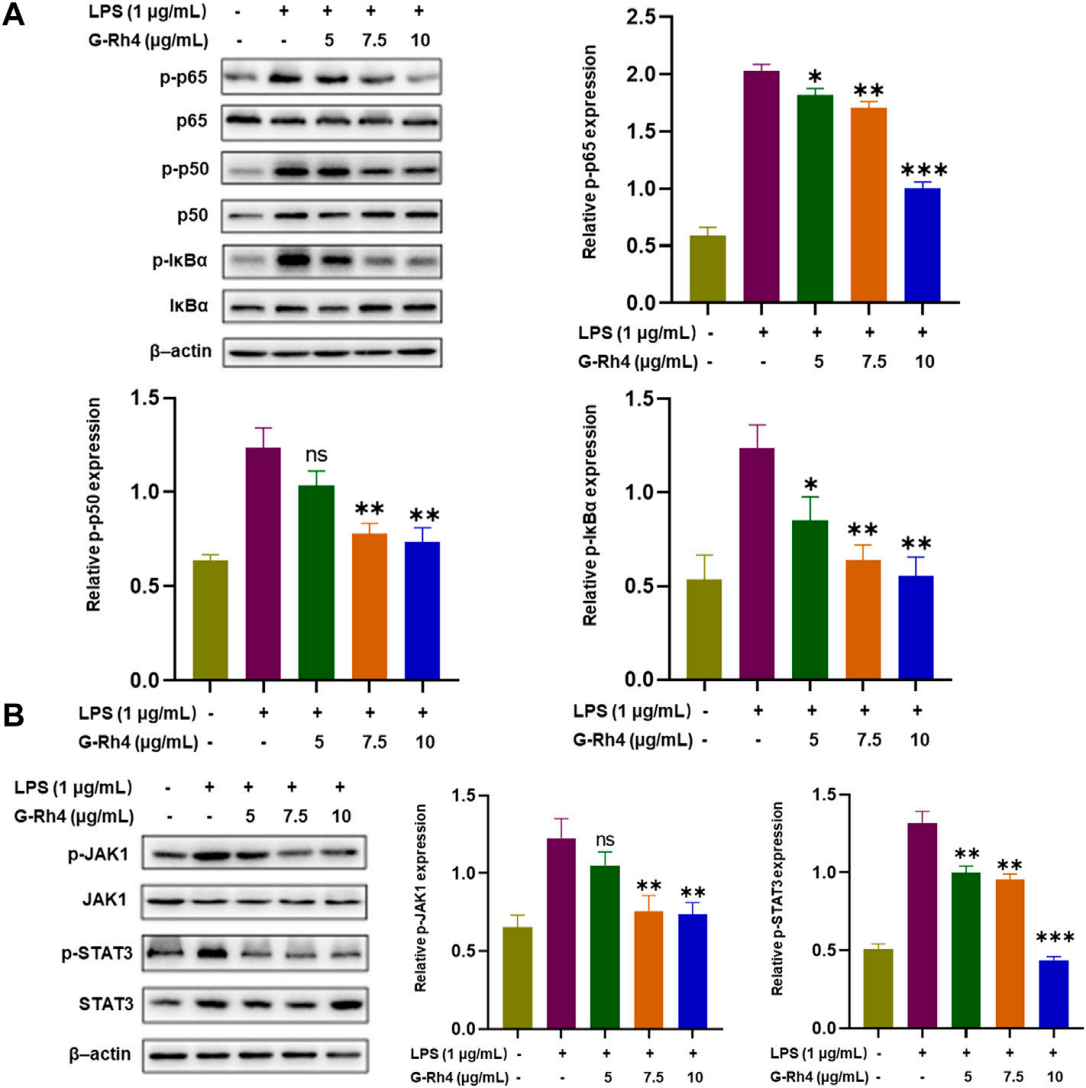
Effect of G-Rh4 on the NF-κB and STAT-3 activation in RAW264.7 cells. **(A)** Determination of the p-IκBα (Ser32), p-p65 (ser536), and p-p50 (ser337) protein using Western blot analysis. **(B)** Effect of G-Rh4 on the STAT-3 activation in RAW264.7 cells. Data were presented as the mean ± SD values performed in triplicate (*p < 0.05, **p < 0.01, ***p < 0.001 vs. LPS group).

NF-κB and STAT3 are transcriptional regulators involved in a series of critical signaling pathways, which plays a key role in the inflammatory response. Nuclear translocation of the NF-κB p50-p65 heterodimer is essential for NF-κB signaling. To further reveal the action mechanisms of G-Rh4, we investigated the effect of G-Rh4 on the expression and activation of p50-p65 and IκBα by Western blot analysis (Figure 6A). The results showed that G-Rh4 effectively inhibited LPS-induced phosphorylation of p50, p65, and IκBα in RAW264.7 cells. According to our network pharmacological analysis, STAT3 is also the main hub gene of G-Rh4 against inflammation, like the main inflammatory factors previously investigated. The Western blot analysis showed that the levels of p-JAK1/JAK1 and p-STAT3/STAT3 were significantly downregulated with the treatment of G-Rh4 (Figure 6B). With the increase of G-Rh4 concentration (5, 7.5, and 10 μg/ml), the phosphorylation levels of JAK1 and STAT3 protein decreased evidently (*p* < 0.01, *p* < 0.001). These results suggest that the anti-inflammatory effect of G-Rh4 can be mediated by the regulation of the NF-kB and STAT3 pathway.

## Discussion

Inflammation is a specific defense response to physical injury or infection. The persistence of inflammation, however, leads to the occurrence and development of several refractory diseases, such as cardiovascular disease, cancer, diabetes mellitus, chronic kidney disease, non-alcoholic fatty liver disease, and autoimmune and neurodegenerative disorders. Therefore, the extensive study of the inflammation pathways and the development of effective inflammation-modulating drugs are the most challenging area in the field of life science and pharmacology. At present, traditional Chinese medicine has become an emerging candidate for the treatment of inflammation because of its potent efficacy and fewer side effects (Fan et al., 2021)**.**

Network pharmacology, from the perspective of system-level and biological networks, analyzes the molecular relationship between drugs and targets, reveals the systematic pharmacological mechanism of drugs, and guides the development of new drugs and clinical diagnosis and treatment. It is reported that the existing differences in data collection and target prediction in network pharmacology analysis result in the loss of some target genes (Luo et al., 2021). Since some target genes may not be included in the public database, we explored multiple databases for G-Rh4 and inflammation-related target genes. A total of 58 anti-inflammatory common targets of G-Rh4 were collected to analyze these common targets by hub gene analysis and GO and KEGG enrichment analysis. Our results showed that the anti-inflammatory effect of G-Rh4 was deeply related to the main target factors including TNF-α, IL-6, IL-1β, and STAT-3 and their related metabolic pathways, and the molecular docking analysis confirmed that G-Rh4 had a high binding possibility with these target factors. VEGFA is also the main target factor related to the anti-inflammatory effect in the network pharmacological analysis, but the possibility of binding with G-Rh4 was not recognized in molecular docking simulation (the experimental result has not shown).

It is reported that a prolonged inflammatory state is harmful to health, and long-term macrophages in tissues activate the abnormal expression of inflammatory factors such as TNF-α, IL-6, IL-1β, NO, and Prostaglandin E2, which can stimulate the inflammatory signaling pathway, eventually leading to chronic low-grade inflammation (Huang et al., 2017). These mediators stimulate the innate immune response, but their overexpression may cause endotoxemia, leading to tissue injury, organ failure, shock, and even death. Regulating the expression of these inflammatory mediators plays a key role in the treatment of inflammatory diseases (Joh et al., 2011). We confirmed the inhibitory effect of G-Rh4 on the expression of the primary target factors through LPS-stimulated mouse RAW264.7 cells. The *in vitro* inflammatory response model established by LPS-stimulated RAW264.7 macrophages is a widely used cell model for screening anti-inflammatory drugs. LPS mainly induces the synthesis and release of a variety of inflammatory mediators through the activation of nuclear transcription factors κB (NF-κB), JAK/STAT, MAPKs, phosphorylated ERK, phosphorylated JNK, and phosphorylated p38 signaling pathways (Liu et al., 2019)**.** Our results indicated that LPS significantly increased mRNA and protein levels of TNF-α, IL-6, and IL-1β. In addition, we also confirmed using ELISAs and RT-PCR methods that G-Rh4 brings about the anti-inflammatory effects by decreasing TNF-α, IL-6, and IL-1β expression levels in LPS-activated RAW264.7 cells.

As reported previously, NO is an important inflammatory signal molecule in the pathogenesis of inflammation, which reacts with superoxide free radicals to produce peroxynitrite ions, resulting in various inflammatory states (Theofilis et al., 2021). Inducible nitric oxide synthase (iNOS) is a regulator of NO synthesis, and it plays a regulatory role in the production of proinflammatory mediators. In addition, inflammation is directly related to arachidonic acid metabolism (Wang T. et al., 2019), under the action of COX-2, and arachidonic acid is transformed into prostaglandin E2(PGE 2) through several enzymatic reactions. PGE_2_ can not only induce the inflammatory cells to release the chemokines and recruit the inflammatory cell movement but also cooperate with lipopolysaccharide to induce the expression of IL-6 and IL-1 in macrophages (Oshima et al., 2011)**.** It can also cooperate with IL-12 to promote the differentiation of naive T cells into helper T cell 1 (Yao et al., 2009). Since COX-2 can quickly respond to a series of proinflammatory mediators and cytokines, it has been considered for a long time to play an important role in the pathological process of inflammation. Moreover, GO analysis of network pharmacology revealed that the target genes of G-Rh4 against inflammation were involved in the “nitric oxide synthase regulator activity” in both biological process (BP) analysis and Molecular Function (MF) analysis. The results in LPS-activated RAW264.7 cells showed that G-Rh4 significantly reduced the release of NO and PGE_2_ as well as the expression of elevated iNOS and COX-2, indicating that network pharmacology might be an effective approach to identify the key target and action pathway of G-Rh4 against inflammation.

In addition, KEGG pathway enrichment analysis pointed out that the intersection target genes of G-Rh4 and inflammation were closely related to the NF-κB and JAK-STAT signaling pathways, the including TNF signaling pathway. Nuclear factor-kappa B(NF-κB) is a protein complex responsible for DNA transcription, cytokine secretion, and cell survival (Wu et al., 2016), and its abnormal signaling pathway is associated with some chronic inflammatory diseases such as inflammatory bowel disease, sepsis, arthritis, and atherosclerosis (Theofilis et al., 2021). NF-κB (p50-p65) is constitutively present in the cytoplasm of RAW264.7cells and is sequestered by inhibitory protein IκBα. In the inflammatory state, the activation phosphorylation of p65 **(**ser536**)** and p50 **(**ser337**)** leads to the activation of NF-κB and the expression of a series of downstream inflammatory factors, such as IL-1β and IL-6. STAT3 (signal transducer and activator of transcription 3) is a key regulator of the inflammatory response induced by lipopolysaccharide (LPS) (Li et al., 2021), and the JAK-STAT (Janus kinase-signal transducer and activator of transcription) pathway is one of the important inflammatory signal transduction pathways, which can mediate the immune response (Zhao et al., 2021). Taken together, this study reveals that G-Rh4 might exert its anti-inflammatory effects through both NF-κB and STAT3 pathways.

Our study clarified, for the first time, the targets and working pathways of G-Rh4 in the inflammation process through network pharmacological analysis and experimental approaches and puts forward that G-Rh4 can directly bind with key pro-inflammatory cytokines including TNF-α, IL-6, and IL-1β to execute its anti-inflammatory activity. The further study is necessary to evaluate the effectiveness of G-Rh4 and explore the extensive working mechanism of *in vivo* inflammation models. This study provides a scientific basis for further pharmacological research on ginsenoside Rh4, as well as the development of novel anti-inflammatory drugs with high efficacy and few side effects and clinical application.

## Data Availability

The datasets presented in this study can be found in online repositories. The names of the repository/repositories and accession number(s) can be found in the article/Supplementary Material.
