# Supplementary material for: Integrative network pharmacology and experimental verification to reveal the anti-inflammatory mechanism of ginsenoside Rh4
Source: Front Pharmacol. 2022 Aug 31;13:953871. doi: 10.3389/fphar.2022.953871 (PMC9471259; doi:10.3389/fphar.2022.953871)
Supplement: Supplementary file 1 [file Table2.docx]

Supplementary Material

Integrative Network Pharmacology and experimental verification to reveal the anti-inflammatory mechanism of Ginsenoside Rh4

**Supplementary material catalog**

Supplementary Table S1. Primer sequences used for RT-PCR

Supplementary Table S2. The 58 potential targets of G-Rh4 for the treatment of Inflammation

Supplementary Table S3. GO enrichment analysis results

Supplementary Table S4. KEGG enrichment analysis results

**Supplementary Table S1. Primer sequences used for RT-PCR.**

| **Target gene** | **Primer Sequences** |
| --- | --- |
| TNF-α | Forward 5’-GGTGCCTATGTCTCAGCCTCTT-3’  Reverse 5’-GCCATAGAACTGATGAGAGGGAG-3’ |
| IL-6 | Forward 5’-TTCTGCCAGTGCCTCTTTGCTG-3’  Reverse 5’-TACCACTTCACAAGTCGGAGGC-3’ |
| IL-1β | Forward 5’-TGGACCTTCCAGGATGAGGACA-3’  Reverse 5’-GTTCATCTCGGAGCCTGTAGTG-3’ |
| iNOS | Forward 5’-CCCTTCCGAAGTTTCTGGCAGCAG-3’  Reverse 5’-GGCTGTCAGAGCCTCGTGGCTTTGG-3’ |
| COX-2 | Forward 5’-CACTACATCCTGACCCACTT-3’  Reverse 5’-ATGCTCCTGCTTGAGTATGT-3’ |
| GAPDH | Forward 5’-ACTCACGGCAAATTCAACGGCA-3’  Reverse 5’-GACTCCACGACATACTCAGCAC-3’ |

**Supplementary Table S2. The 58 potential targets of G-Rh4 for the treatment of Inflammation**

| **Uniprot ID** | **Full Name** | **Symbol** |
| --- | --- | --- |
| P05231 | Interleukin-6 | IL6 |
| P01375 | Tumor necrosis factor | TNF |
| P01584 | Interleukin-1 beta | IL1B |
| P43405 | Tyrosine-protein kinase SYK | SYK |
| P35354 | Prostaglandin G/H synthase 2 | PTGS2 |
| Q9Y2R2 | Tyrosine-protein phosphatase non-receptor type 22 | PTPN22 |
| P40763 | Signal transducer and activator of transcription 3 | STAT3 |
| P14780 | Matrix metalloproteinase-9 | MMP9 |
| P19838 | Nuclear factor NF-kappa-B p105 subunit | NFKB1 |
| P19320 | Vascular cell adhesion protein 1 | VCAM1 |
| P60568 | Interleukin-2 | IL2 |
| P15692 | Vascular endothelial growth factor A | VEGFA |
| P32246 | C-C chemokine receptor type 1 | CCR1 |
| P23458 | Tyrosine-protein kinase JAK1 | JAK1 |
| P29466 | Caspase-1 | CASP1 |
| P37231 | Peroxisome proliferator-activated receptor gamma | PPARG |
| P08183 | ATP-dependent translocase ABCB1 | ABCB1 |
| P35228 | Nitric oxide synthase, inducible | NOS2 |
| P04083 | Annexin A1 | ANXA1 |
| Q16539 | Mitogen-activated protein kinase 14 | MAPK14 |
| P51677 | C-C chemokine receptor type 3 | CCR3 |
| P04150 | Glucocorticoid receptor | NR3C1 |
| P06858 | Lipoprotein lipase | LPL |
| P13726 | Tissue factor | F3 |
| P00533 | Epidermal growth factor receptor | EGFR |
| Q00653 | Nuclear factor NF-kappa-B p100 subunit | NFKB2 |
| P31749 | RAC-alpha serine/threonine-protein kinase | AKT1 |
| P42336 | Phosphatidylinositol 4,5-bisphosphate 3-kinase catalytic subunit alpha isoform | PIK3CA |
| P11473 | Vitamin D3 receptor | VDR |
| P42574 | Caspase-3 | CASP3 |
| P48736 | Phosphatidylinositol 4,5-bisphosphate 3-kinase catalytic subunit gamma isoform | PIK3CG |
| P08473 | Neprilysin | MME |
| P28482 | Mitogen-activated protein kinase 1 | MAPK1 |
| P07477 | Serine protease 1 | PRSS1 |
| O95342 | Bile salt export pump | ABCB11 |
| Q9Y5Y4 | Prostaglandin D2 receptor 2 | PTGDR2 |
| P45983 | Mitogen-activated protein kinase 8 | MAPK8 |
| P05177 | Cytochrome P450 1A2 | CYP1A2 |
| Q07817 | Bcl-2-like protein 1 | BCL2L1 |
| P05067 | Amyloid-beta precursor protein | APP |
| P03372 | Estrogen receptor | ESR1 |
| P42345 | Serine/threonine-protein kinase mTOR | MTOR |
| P10275 | Androgen receptor | AR |
| P09038 | Fibroblast growth factor 2 | FGF2 |
| Q13085 | Acetyl-CoA carboxylase 1 | ACACA |
| P24941 | Cyclin-dependent kinase 2 | CDK2 |
| Q96EB6 | NAD-dependent protein deacetylase sirtuin-1 | SIRT1 |
| P07900 | Heat shock protein HSP 90-alpha | HSP90AA1 |
| O14684 | Prostaglandin E synthase | PTGES |
| P51449 | Nuclear receptor ROR-gamma | RORC |
| P08842 | Steryl-sulfatase | STS |
| P18031 | Tyrosine-protein phosphatase non-receptor type 1 | PTPN1 |
| O76074 | cGMP-specific 3',5'-cyclic phosphodiesterase | PDE5A |
| P30542 | Adenosine receptor A1 | ADORA1 |
| P0DMS8 | Adenosine receptor A3 | ADORA3 |
| Q9P1W9 | Serine/threonine-protein kinase pim-2 | PIM2 |
| P21453 | Sphingosine 1-phosphate receptor 1 | S1PR1 |
| P08908 | 5-hydroxytryptamine receptor 1A | HTR1A |

**Supplementary Table S3. GO enrichment analysis results**

| **Category** | **Term** | **Count** | **%** | **P Value** |
| --- | --- | --- | --- | --- |
| GOTERM_BP_DIRECT | GO:0010628~positive regulation of gene expression | 17 | 29.310 | 1.04E-12 |
| GOTERM_BP_DIRECT | GO:0032755~positive regulation of interleukin-6 production | 14 | 24.138 | 4.84E-12 |
| GOTERM_BP_DIRECT | GO:0006954~inflammatory response | 15 | 25.862 | 8.98E-12 |
| GOTERM_BP_DIRECT | GO:0031663~lipopolysaccharide-mediated signaling pathway | 9 | 15.517 | 2.68E-11 |
| GOTERM_BP_DIRECT | GO:0045429~positive regulation of nitric oxide biosynthetic process | 8 | 13.793 | 9.44E-11 |
| GOTERM_BP_DIRECT | GO:0051897~positive regulation of protein kinase B signaling | 10 | 17.241 | 1.37E-10 |
| GOTERM_BP_DIRECT | GO:0033138~positive regulation of peptidyl-serine phosphorylation | 9 | 15.517 | 2.59E-10 |
| GOTERM_BP_DIRECT | GO:0045893~positive regulation of transcription, DNA-templated | 20 | 34.483 | 6.30E-10 |
| GOTERM_BP_DIRECT | GO:0008285~negative regulation of cell proliferation | 20 | 34.483 | 9.07E-10 |
| GOTERM_BP_DIRECT | GO:0043066~negative regulation of apoptotic process | 14 | 24.138 | 2.74E-09 |
| GOTERM_BP_DIRECT | GO:0043406~positive regulation of MAP kinase activity | 8 | 13.793 | 3.79E-09 |
| GOTERM_BP_DIRECT | GO:0045944~positive regulation of transcription from RNA polymerase II promoter | 15 | 25.862 | 5.77E-09 |
| GOTERM_BP_DIRECT | GO:0001934~positive regulation of protein phosphorylation | 10 | 17.241 | 8.53E-09 |
| GOTERM_BP_DIRECT | GO:0032496~response to lipopolysaccharide | 9 | 15.517 | 1.90E-08 |
| GOTERM_BP_DIRECT | GO:0010629~negative regulation of gene expression | 8 | 13.793 | 1.95E-08 |
| GOTERM_BP_DIRECT | GO:0032757~positive regulation of interleukin-8 production | 7 | 12.069 | 3.47E-08 |
| GOTERM_BP_DIRECT | GO:0048661~positive regulation of smooth muscle cell proliferation | 6 | 10.345 | 7.90E-08 |
| GOTERM_BP_DIRECT | GO:0001938~positive regulation of endothelial cell proliferation | 7 | 12.069 | 8.39E-08 |
| GOTERM_BP_DIRECT | GO:0007165~signal transduction | 12 | 20.690 | 8.67E-08 |
| GOTERM_BP_DIRECT | GO:0071276~cellular response to cadmium ion | 6 | 10.345 | 1.35E-07 |
| GOTERM_BP_DIRECT | GO:0071260~cellular response to mechanical stimulus | 7 | 12.069 | 1.80E-07 |
| GOTERM_BP_DIRECT | GO:0060252~positive regulation of glial cell proliferation | 5 | 8.621 | 2.09E-07 |
| GOTERM_BP_DIRECT | GO:0008284~positive regulation of cell proliferation | 12 | 20.690 | 4.12E-07 |
| GOTERM_BP_DIRECT | GO:0043065~positive regulation of apoptotic process | 10 | 17.241 | 5.08E-07 |
| GOTERM_BP_DIRECT | GO:0050729~positive regulation of inflammatory response | 7 | 12.069 | 6.06E-07 |
| GOTERM_BP_DIRECT | GO:0030335~positive regulation of cell migration | 9 | 15.517 | 8.56E-07 |
| GOTERM_BP_DIRECT | GO:0032731~positive regulation of interleukin-1 beta production | 6 | 10.345 | 1.30E-06 |
| GOTERM_BP_DIRECT | GO:0051091~positive regulation of sequence-specific DNA binding transcription factor activity | 7 | 12.069 | 1.42E-06 |
| GOTERM_BP_DIRECT | GO:0071222~cellular response to lipopolysaccharide | 8 | 13.793 | 1.42E-06 |
| GOTERM_BP_DIRECT | GO:0007568~aging | 8 | 13.793 | 1.47E-06 |
| GOTERM_BP_DIRECT | GO:0006935~chemotaxis | 7 | 12.069 | 1.72E-06 |
| GOTERM_BP_DIRECT | GO:0006468~protein phosphorylation | 11 | 18.966 | 2.18E-06 |
| GOTERM_BP_DIRECT | GO:0035924~cellular response to vascular endothelial growth factor stimulus | 5 | 8.621 | 3.06E-06 |
| GOTERM_BP_DIRECT | GO:0034614~cellular response to reactive oxygen species | 5 | 8.621 | 4.32E-06 |
| GOTERM_BP_DIRECT | GO:0019221~cytokine-mediated signaling pathway | 7 | 12.069 | 5.87E-06 |
| GOTERM_BP_DIRECT | GO:0043410~positive regulation of MAPK cascade | 7 | 12.069 | 7.61E-06 |
| GOTERM_BP_DIRECT | GO:0045766~positive regulation of angiogenesis | 7 | 12.069 | 8.17E-06 |
| GOTERM_BP_DIRECT | GO:0001525~angiogenesis | 8 | 13.793 | 8.31E-06 |
| GOTERM_BP_DIRECT | GO:1902895~positive regulation of pri-miRNA transcription from RNA polymerase II promoter | 5 | 8.621 | 1.05E-05 |
| GOTERM_BP_DIRECT | GO:0018105~peptidyl-serine phosphorylation | 7 | 12.069 | 1.23E-05 |
| GOTERM_BP_DIRECT | GO:0001666~response to hypoxia | 7 | 12.069 | 1.54E-05 |
| GOTERM_BP_DIRECT | GO:0010507~negative regulation of autophagy | 5 | 8.621 | 1.59E-05 |
| GOTERM_BP_DIRECT | GO:0048143~astrocyte activation | 4 | 6.897 | 1.65E-05 |
| GOTERM_BP_DIRECT | GO:0000122~negative regulation of transcription from RNA polymerase II promoter | 13 | 22.414 | 1.89E-05 |
| GOTERM_BP_DIRECT | GO:0043280~positive regulation of cysteine-type endopeptidase activity involved in apoptotic process | 5 | 8.621 | 1.99E-05 |
| GOTERM_BP_DIRECT | GO:0032355~response to estradiol | 6 | 10.345 | 2.14E-05 |
| GOTERM_BP_DIRECT | GO:0006979~response to oxidative stress | 6 | 10.345 | 2.75E-05 |
| GOTERM_BP_DIRECT | GO:0007611~learning or memory | 5 | 8.621 | 3.45E-05 |
| GOTERM_BP_DIRECT | GO:0071456~cellular response to hypoxia | 6 | 10.345 | 4.36E-05 |
| GOTERM_BP_DIRECT | GO:0070374~positive regulation of ERK1 and ERK2 cascade | 7 | 12.069 | 5.54E-05 |
| GOTERM_BP_DIRECT | GO:0071356~cellular response to tumor necrosis factor | 6 | 10.345 | 6.19E-05 |
| GOTERM_BP_DIRECT | GO:0031622~positive regulation of fever generation | 3 | 5.172 | 8.65E-05 |
| GOTERM_BP_DIRECT | GO:0032729~positive regulation of interferon-gamma production | 5 | 8.621 | 8.96E-05 |
| GOTERM_BP_DIRECT | GO:0007204~positive regulation of cytosolic calcium ion concentration | 6 | 10.345 | 9.70E-05 |
| GOTERM_BP_DIRECT | GO:0042327~positive regulation of phosphorylation | 4 | 6.897 | 1.06E-04 |
| GOTERM_BP_DIRECT | GO:0019722~calcium-mediated signaling | 5 | 8.621 | 1.25E-04 |
| GOTERM_BP_DIRECT | GO:0050727~regulation of inflammatory response | 5 | 8.621 | 1.42E-04 |
| GOTERM_BP_DIRECT | GO:0030324~lung development | 5 | 8.621 | 1.49E-04 |
| GOTERM_BP_DIRECT | GO:0090050~positive regulation of cell migration involved in sprouting angiogenesis | 4 | 6.897 | 1.53E-04 |
| GOTERM_BP_DIRECT | GO:2001240~negative regulation of extrinsic apoptotic signaling pathway in absence of ligand | 4 | 6.897 | 1.66E-04 |
| GOTERM_BP_DIRECT | GO:0010573~vascular endothelial growth factor production | 3 | 5.172 | 1.81E-04 |
| GOTERM_BP_DIRECT | GO:0031394~positive regulation of prostaglandin biosynthetic process | 3 | 5.172 | 1.81E-04 |
| GOTERM_BP_DIRECT | GO:0042493~response to drug | 7 | 12.069 | 2.29E-04 |
| GOTERM_BP_DIRECT | GO:0043491~protein kinase B signaling | 4 | 6.897 | 2.46E-04 |
| GOTERM_BP_DIRECT | GO:0032760~positive regulation of tumor necrosis factor production | 5 | 8.621 | 2.79E-04 |
| GOTERM_BP_DIRECT | GO:0042307~positive regulation of protein import into nucleus | 4 | 6.897 | 2.83E-04 |
| GOTERM_BP_DIRECT | GO:0006915~apoptotic process | 9 | 15.517 | 3.08E-04 |
| GOTERM_BP_DIRECT | GO:0032722~positive regulation of chemokine production | 4 | 6.897 | 3.24E-04 |
| GOTERM_BP_DIRECT | GO:0050796~regulation of insulin secretion | 4 | 6.897 | 3.46E-04 |
| GOTERM_BP_DIRECT | GO:0060397~JAK-STAT cascade involved in growth hormone signaling pathway | 3 | 5.172 | 3.85E-04 |
| GOTERM_BP_DIRECT | GO:0010888~negative regulation of lipid storage | 3 | 5.172 | 3.85E-04 |
| GOTERM_BP_DIRECT | GO:0043536~positive regulation of blood vessel endothelial cell migration | 4 | 6.897 | 4.43E-04 |
| GOTERM_BP_DIRECT | GO:0030518~intracellular steroid hormone receptor signaling pathway | 3 | 5.172 | 4.70E-04 |
| GOTERM_BP_DIRECT | GO:0043276~anoikis | 3 | 5.172 | 4.70E-04 |
| GOTERM_BP_DIRECT | GO:0042981~regulation of apoptotic process | 6 | 10.345 | 4.92E-04 |
| GOTERM_BP_DIRECT | GO:0016310~phosphorylation | 5 | 8.621 | 5.55E-04 |
| GOTERM_BP_DIRECT | GO:0019233~sensory perception of pain | 4 | 6.897 | 5.56E-04 |
| GOTERM_BP_DIRECT | GO:0050927~positive regulation of positive chemotaxis | 3 | 5.172 | 5.63E-04 |
| GOTERM_BP_DIRECT | GO:0098586~cellular response to virus | 4 | 6.897 | 5.87E-04 |
| GOTERM_BP_DIRECT | GO:0071407~cellular response to organic cyclic compound | 4 | 6.897 | 5.87E-04 |
| GOTERM_BP_DIRECT | GO:0071230~cellular response to amino acid stimulus | 4 | 6.897 | 5.87E-04 |
| GOTERM_BP_DIRECT | GO:0007193~adenylate cyclase-inhibiting G-protein coupled receptor signaling pathway | 4 | 6.897 | 5.87E-04 |
| GOTERM_BP_DIRECT | GO:0014823~response to activity | 4 | 6.897 | 5.87E-04 |
| GOTERM_BP_DIRECT | GO:0045471~response to ethanol | 5 | 8.621 | 5.88E-04 |
| GOTERM_BP_DIRECT | GO:0003376~sphingosine-1-phosphate signaling pathway | 3 | 5.172 | 7.74E-04 |
| GOTERM_BP_DIRECT | GO:0006006~glucose metabolic process | 4 | 6.897 | 8.74E-04 |
| GOTERM_BP_DIRECT | GO:0030168~platelet activation | 4 | 6.897 | 8.74E-04 |
| GOTERM_BP_DIRECT | GO:0016242~negative regulation of macroautophagy | 3 | 5.172 | 8.91E-04 |
| GOTERM_BP_DIRECT | GO:0070102~interleukin-6-mediated signaling pathway | 3 | 5.172 | 8.91E-04 |
| GOTERM_BP_DIRECT | GO:1901215~negative regulation of neuron death | 4 | 6.897 | 9.15E-04 |
| GOTERM_BP_DIRECT | GO:0042102~positive regulation of T cell proliferation | 4 | 6.897 | 9.15E-04 |
| GOTERM_BP_DIRECT | GO:0051146~striated muscle cell differentiation | 3 | 5.172 | 0.001016196 |
| GOTERM_BP_DIRECT | GO:0010595~positive regulation of endothelial cell migration | 4 | 6.897 | 0.001045315 |
| GOTERM_BP_DIRECT | GO:0051384~response to glucocorticoid | 4 | 6.897 | 0.001045315 |
| GOTERM_BP_DIRECT | GO:0043154~negative regulation of cysteine-type endopeptidase activity involved in apoptotic process | 4 | 6.897 | 0.001091264 |
| GOTERM_BP_DIRECT | GO:0007623~circadian rhythm | 4 | 6.897 | 0.001091264 |
| GOTERM_BP_DIRECT | GO:0043627~response to estrogen | 4 | 6.897 | 0.00113847 |
| GOTERM_BP_DIRECT | GO:0060749~mammary gland alveolus development | 3 | 5.172 | 0.00114949 |
| GOTERM_BP_DIRECT | GO:0071380~cellular response to prostaglandin E stimulus | 3 | 5.172 | 0.00114949 |
| GOTERM_BP_DIRECT | GO:0050995~negative regulation of lipid catabolic process | 3 | 5.172 | 0.00114949 |
| GOTERM_BP_DIRECT | GO:1901224~positive regulation of NIK/NF-kappaB signaling | 4 | 6.897 | 0.001186949 |
| GOTERM_BP_DIRECT | GO:0018107~peptidyl-threonine phosphorylation | 4 | 6.897 | 0.001236711 |
| GOTERM_BP_DIRECT | GO:0002526~acute inflammatory response | 3 | 5.172 | 0.001290708 |
| GOTERM_BP_DIRECT | GO:0035994~response to muscle stretch | 3 | 5.172 | 0.001290708 |
| GOTERM_BP_DIRECT | GO:0051044~positive regulation of membrane protein ectodomain proteolysis | 3 | 5.172 | 0.001290708 |
| GOTERM_BP_DIRECT | GO:0051092~positive regulation of NF-kappaB transcription factor activity | 5 | 8.621 | 0.001312102 |
| GOTERM_BP_DIRECT | GO:2000573~positive regulation of DNA biosynthetic process | 3 | 5.172 | 0.001439804 |
| GOTERM_BP_DIRECT | GO:0048511~rhythmic process | 4 | 6.897 | 0.001562978 |
| GOTERM_BP_DIRECT | GO:0050730~regulation of peptidyl-tyrosine phosphorylation | 3 | 5.172 | 0.00159673 |
| GOTERM_BP_DIRECT | GO:0032930~positive regulation of superoxide anion generation | 3 | 5.172 | 0.001761441 |
| GOTERM_BP_DIRECT | GO:0002042~cell migration involved in sprouting angiogenesis | 3 | 5.172 | 0.001761441 |
| GOTERM_BP_DIRECT | GO:0014068~positive regulation of phosphatidylinositol 3-kinase signaling | 4 | 6.897 | 0.001872474 |
| GOTERM_BP_DIRECT | GO:0030225~macrophage differentiation | 3 | 5.172 | 0.00193389 |
| GOTERM_BP_DIRECT | GO:0046777~protein autophosphorylation | 5 | 8.621 | 0.002019925 |
| GOTERM_BP_DIRECT | GO:0050768~negative regulation of neurogenesis | 3 | 5.172 | 0.002301821 |
| GOTERM_BP_DIRECT | GO:0097194~execution phase of apoptosis | 3 | 5.172 | 0.002497212 |
| GOTERM_BP_DIRECT | GO:0040014~regulation of multicellular organism growth | 3 | 5.172 | 0.002497212 |
| GOTERM_BP_DIRECT | GO:0050731~positive regulation of peptidyl-tyrosine phosphorylation | 4 | 6.897 | 0.002681114 |
| GOTERM_BP_DIRECT | GO:0050829~defense response to Gram-negative bacterium | 4 | 6.897 | 0.002681114 |
| GOTERM_BP_DIRECT | GO:0051000~positive regulation of nitric-oxide synthase activity | 3 | 5.172 | 0.002700158 |
| GOTERM_BP_DIRECT | GO:0046697~decidualization | 3 | 5.172 | 0.002910616 |
| GOTERM_BP_DIRECT | GO:1900017~positive regulation of cytokine production involved in inflammatory response | 3 | 5.172 | 0.00312854 |
| GOTERM_BP_DIRECT | GO:2001243~negative regulation of intrinsic apoptotic signaling pathway | 3 | 5.172 | 0.00312854 |
| GOTERM_BP_DIRECT | GO:0042060~wound healing | 4 | 6.897 | 0.003292242 |
| GOTERM_BP_DIRECT | GO:0097192~extrinsic apoptotic signaling pathway in absence of ligand | 3 | 5.172 | 0.003353885 |
| GOTERM_BP_DIRECT | GO:0070372~regulation of ERK1 and ERK2 cascade | 3 | 5.172 | 0.003353885 |
| GOTERM_BP_DIRECT | GO:1903672~positive regulation of sprouting angiogenesis | 3 | 5.172 | 0.003353885 |
| GOTERM_BP_DIRECT | GO:0030154~cell differentiation | 8 | 13.793 | 0.003420237 |
| GOTERM_BP_DIRECT | GO:0010575~positive regulation of vascular endothelial growth factor production | 3 | 5.172 | 0.003586607 |
| GOTERM_BP_DIRECT | GO:0046677~response to antibiotic | 3 | 5.172 | 0.003826662 |
| GOTERM_BP_DIRECT | GO:0060045~positive regulation of cardiac muscle cell proliferation | 3 | 5.172 | 0.004328593 |
| GOTERM_BP_DIRECT | GO:0042593~glucose homeostasis | 4 | 6.897 | 0.00475446 |
| GOTERM_BP_DIRECT | GO:0007267~cell-cell signaling | 5 | 8.621 | 0.004787194 |
| GOTERM_BP_DIRECT | GO:0030522~intracellular receptor signaling pathway | 3 | 5.172 | 0.004859327 |
| GOTERM_BP_DIRECT | GO:0009410~response to xenobiotic stimulus | 5 | 8.621 | 0.005322026 |
| GOTERM_BP_DIRECT | GO:0014065~phosphatidylinositol 3-kinase signaling | 3 | 5.172 | 0.005708676 |
| GOTERM_BP_DIRECT | GO:0045907~positive regulation of vasoconstriction | 3 | 5.172 | 0.005708676 |
| GOTERM_BP_DIRECT | GO:0001890~placenta development | 3 | 5.172 | 0.005708676 |
| GOTERM_BP_DIRECT | GO:0042789~mRNA transcription from RNA polymerase II promoter | 3 | 5.172 | 0.005708676 |
| GOTERM_BP_DIRECT | GO:0010749~regulation of nitric oxide mediated signal transduction | 2 | 3.448 | 0.005942129 |
| GOTERM_BP_DIRECT | GO:0007281~germ cell development | 3 | 5.172 | 0.006005819 |
| GOTERM_BP_DIRECT | GO:0035094~response to nicotine | 3 | 5.172 | 0.006309904 |
| GOTERM_BP_DIRECT | GO:0090398~cellular senescence | 3 | 5.172 | 0.006309904 |
| GOTERM_BP_DIRECT | GO:0097009~energy homeostasis | 3 | 5.172 | 0.006309904 |
| GOTERM_BP_DIRECT | GO:0070555~response to interleukin-1 | 3 | 5.172 | 0.006309904 |
| GOTERM_BP_DIRECT | GO:0097421~liver regeneration | 3 | 5.172 | 0.006309904 |
| GOTERM_BP_DIRECT | GO:0043537~negative regulation of blood vessel endothelial cell migration | 3 | 5.172 | 0.006309904 |
| GOTERM_BP_DIRECT | GO:0007186~G-protein coupled receptor signaling pathway | 9 | 15.517 | 0.006497793 |
| GOTERM_BP_DIRECT | GO:0032733~positive regulation of interleukin-10 production | 3 | 5.172 | 0.006620889 |
| GOTERM_BP_DIRECT | GO:0009409~response to cold | 3 | 5.172 | 0.006620889 |
| GOTERM_BP_DIRECT | GO:0010468~regulation of gene expression | 5 | 8.621 | 0.006784536 |
| GOTERM_BP_DIRECT | GO:0007566~embryo implantation | 3 | 5.172 | 0.006938731 |
| GOTERM_BP_DIRECT | GO:1901216~positive regulation of neuron death | 3 | 5.172 | 0.006938731 |
| GOTERM_BP_DIRECT | GO:0043507~positive regulation of JUN kinase activity | 3 | 5.172 | 0.00759482 |
| GOTERM_BP_DIRECT | GO:0034198~cellular response to amino acid starvation | 3 | 5.172 | 0.007932983 |
| GOTERM_BP_DIRECT | GO:0051402~neuron apoptotic process | 3 | 5.172 | 0.007932983 |
| GOTERM_BP_DIRECT | GO:0043407~negative regulation of MAP kinase activity | 3 | 5.172 | 0.007932983 |
| GOTERM_BP_DIRECT | GO:1904646~cellular response to beta-amyloid | 3 | 5.172 | 0.008629339 |
| GOTERM_BP_DIRECT | GO:0008630~intrinsic apoptotic signaling pathway in response to DNA damage | 3 | 5.172 | 0.008629339 |
| GOTERM_BP_DIRECT | GO:0060559~positive regulation of calcidiol 1-monooxygenase activity | 2 | 3.448 | 0.008900171 |
| GOTERM_BP_DIRECT | GO:0031281~positive regulation of cyclase activity | 2 | 3.448 | 0.008900171 |
| GOTERM_BP_DIRECT | GO:2000635~negative regulation of primary miRNA processing | 2 | 3.448 | 0.008900171 |
| GOTERM_BP_DIRECT | GO:0070141~response to UV-A | 2 | 3.448 | 0.008900171 |
| GOTERM_BP_DIRECT | GO:0051781~positive regulation of cell division | 3 | 5.172 | 0.008987449 |
| GOTERM_BP_DIRECT | GO:0007173~epidermal growth factor receptor signaling pathway | 3 | 5.172 | 0.008987449 |
| GOTERM_BP_DIRECT | GO:0009408~response to heat | 3 | 5.172 | 0.008987449 |
| GOTERM_BP_DIRECT | GO:0010718~positive regulation of epithelial to mesenchymal transition | 3 | 5.172 | 0.008987449 |
| GOTERM_BP_DIRECT | GO:1900087~positive regulation of G1/S transition of mitotic cell cycle | 3 | 5.172 | 0.009352126 |
| GOTERM_BP_DIRECT | GO:0007254~JNK cascade | 3 | 5.172 | 0.009723328 |
| GOTERM_BP_DIRECT | GO:0034097~response to cytokine | 3 | 5.172 | 0.010101016 |
| GOTERM_BP_DIRECT | GO:0045600~positive regulation of fat cell differentiation | 3 | 5.172 | 0.010101016 |
| GOTERM_BP_DIRECT | GO:1904707~positive regulation of vascular smooth muscle cell proliferation | 3 | 5.172 | 0.010101016 |
| GOTERM_BP_DIRECT | GO:0050890~cognition | 3 | 5.172 | 0.010101016 |
| GOTERM_BP_DIRECT | GO:0035556~intracellular signal transduction | 6 | 10.345 | 0.010191457 |
| GOTERM_BP_DIRECT | GO:0006357~regulation of transcription from RNA polymerase II promoter | 12 | 20.690 | 0.010553179 |
| GOTERM_BP_DIRECT | GO:0030198~extracellular matrix organization | 4 | 6.897 | 0.011633246 |
| GOTERM_BP_DIRECT | GO:0016241~regulation of macroautophagy | 3 | 5.172 | 0.011675813 |
| GOTERM_BP_DIRECT | GO:0055088~lipid homeostasis | 3 | 5.172 | 0.011675813 |
| GOTERM_BP_DIRECT | GO:0031334~positive regulation of protein complex assembly | 3 | 5.172 | 0.011675813 |
| GOTERM_BP_DIRECT | GO:0002366~leukocyte activation involved in immune response | 2 | 3.448 | 0.011849565 |
| GOTERM_BP_DIRECT | GO:0002685~regulation of leukocyte migration | 2 | 3.448 | 0.011849565 |
| GOTERM_BP_DIRECT | GO:0060745~mammary gland branching involved in pregnancy | 2 | 3.448 | 0.011849565 |
| GOTERM_BP_DIRECT | GO:0060571~morphogenesis of an epithelial fold | 2 | 3.448 | 0.011849565 |
| GOTERM_BP_DIRECT | GO:0090400~stress-induced premature senescence | 2 | 3.448 | 0.011849565 |
| GOTERM_BP_DIRECT | GO:0030730~sequestering of triglyceride | 2 | 3.448 | 0.011849565 |
| GOTERM_BP_DIRECT | GO:0042127~regulation of cell proliferation | 4 | 6.897 | 0.012236318 |
| GOTERM_BP_DIRECT | GO:0007166~cell surface receptor signaling pathway | 5 | 8.621 | 0.012288179 |
| GOTERM_BP_DIRECT | GO:0045599~negative regulation of fat cell differentiation | 3 | 5.172 | 0.01292304 |
| GOTERM_BP_DIRECT | GO:1903078~positive regulation of protein localization to plasma membrane | 3 | 5.172 | 0.01292304 |
| GOTERM_BP_DIRECT | GO:0031532~actin cytoskeleton reorganization | 3 | 5.172 | 0.013351168 |
| GOTERM_BP_DIRECT | GO:0043525~positive regulation of neuron apoptotic process | 3 | 5.172 | 0.013785423 |
| GOTERM_BP_DIRECT | GO:0006955~immune response | 6 | 10.345 | 0.013796283 |
| GOTERM_BP_DIRECT | GO:0055118~negative regulation of cardiac muscle contraction | 2 | 3.448 | 0.014790336 |
| GOTERM_BP_DIRECT | GO:1905278~positive regulation of epithelial tube formation | 2 | 3.448 | 0.014790336 |
| GOTERM_BP_DIRECT | GO:0090335~regulation of brown fat cell differentiation | 2 | 3.448 | 0.014790336 |
| GOTERM_BP_DIRECT | GO:0046883~regulation of hormone secretion | 2 | 3.448 | 0.014790336 |
| GOTERM_BP_DIRECT | GO:0097190~apoptotic signaling pathway | 3 | 5.172 | 0.015124563 |
| GOTERM_BP_DIRECT | GO:0050679~positive regulation of epithelial cell proliferation | 3 | 5.172 | 0.015582939 |
| GOTERM_BP_DIRECT | GO:0007187~G-protein coupled receptor signaling pathway, coupled to cyclic nucleotide second messenger | 3 | 5.172 | 0.01604725 |
| GOTERM_BP_DIRECT | GO:0042752~regulation of circadian rhythm | 3 | 5.172 | 0.01604725 |
| GOTERM_BP_DIRECT | GO:0006909~phagocytosis | 3 | 5.172 | 0.016993524 |
| GOTERM_BP_DIRECT | GO:0035690~cellular response to drug | 3 | 5.172 | 0.016993524 |
| GOTERM_BP_DIRECT | GO:1903223~positive regulation of oxidative stress-induced neuron death | 2 | 3.448 | 0.017722507 |
| GOTERM_BP_DIRECT | GO:0051712~positive regulation of killing of cells of other organism | 2 | 3.448 | 0.017722507 |
| GOTERM_BP_DIRECT | GO:0031998~regulation of fatty acid beta-oxidation | 2 | 3.448 | 0.017722507 |
| GOTERM_BP_DIRECT | GO:0006706~steroid catabolic process | 2 | 3.448 | 0.017722507 |
| GOTERM_BP_DIRECT | GO:1903169~regulation of calcium ion transmembrane transport | 2 | 3.448 | 0.017722507 |
| GOTERM_BP_DIRECT | GO:0042531~positive regulation of tyrosine phosphorylation of STAT protein | 3 | 5.172 | 0.01796308 |
| GOTERM_BP_DIRECT | GO:0060326~cell chemotaxis | 3 | 5.172 | 0.01895562 |
| GOTERM_BP_DIRECT | GO:0043123~positive regulation of I-kappaB kinase/NF-kappaB signaling | 4 | 6.897 | 0.019252676 |
| GOTERM_BP_DIRECT | GO:0070301~cellular response to hydrogen peroxide | 3 | 5.172 | 0.019970846 |
| GOTERM_BP_DIRECT | GO:0016485~protein processing | 3 | 5.172 | 0.020486874 |
| GOTERM_BP_DIRECT | GO:1903721~positive regulation of I-kappaB phosphorylation | 2 | 3.448 | 0.020646105 |
| GOTERM_BP_DIRECT | GO:1900122~positive regulation of receptor binding | 2 | 3.448 | 0.020646105 |
| GOTERM_BP_DIRECT | GO:0034121~regulation of toll-like receptor signaling pathway | 2 | 3.448 | 0.020646105 |
| GOTERM_BP_DIRECT | GO:0043457~regulation of cellular respiration | 2 | 3.448 | 0.020646105 |
| GOTERM_BP_DIRECT | GO:0050996~positive regulation of lipid catabolic process | 2 | 3.448 | 0.020646105 |
| GOTERM_BP_DIRECT | GO:0072540~T-helper 17 cell lineage commitment | 2 | 3.448 | 0.020646105 |
| GOTERM_BP_DIRECT | GO:0007584~response to nutrient | 3 | 5.172 | 0.021535578 |
| GOTERM_BP_DIRECT | GO:0032868~response to insulin | 3 | 5.172 | 0.02206818 |
| GOTERM_BP_DIRECT | GO:0044321~response to leptin | 2 | 3.448 | 0.023561153 |
| GOTERM_BP_DIRECT | GO:0033210~leptin-mediated signaling pathway | 2 | 3.448 | 0.023561153 |
| GOTERM_BP_DIRECT | GO:2000270~negative regulation of fibroblast apoptotic process | 2 | 3.448 | 0.023561153 |
| GOTERM_BP_DIRECT | GO:0002674~negative regulation of acute inflammatory response | 2 | 3.448 | 0.023561153 |
| GOTERM_BP_DIRECT | GO:0007155~cell adhesion | 6 | 10.345 | 0.023896244 |
| GOTERM_BP_DIRECT | GO:0007399~nervous system development | 5 | 8.621 | 0.024241233 |
| GOTERM_BP_DIRECT | GO:0045727~positive regulation of translation | 3 | 5.172 | 0.024252757 |
| GOTERM_BP_DIRECT | GO:0030593~neutrophil chemotaxis | 3 | 5.172 | 0.024252757 |
| GOTERM_BP_DIRECT | GO:0006919~activation of cysteine-type endopeptidase activity involved in apoptotic process | 3 | 5.172 | 0.024812264 |
| GOTERM_BP_DIRECT | GO:0045822~negative regulation of heart contraction | 2 | 3.448 | 0.026467676 |
| GOTERM_BP_DIRECT | GO:0050999~regulation of nitric-oxide synthase activity | 2 | 3.448 | 0.026467676 |
| GOTERM_BP_DIRECT | GO:0072089~stem cell proliferation | 2 | 3.448 | 0.026467676 |
| GOTERM_BP_DIRECT | GO:0071316~cellular response to nicotine | 2 | 3.448 | 0.026467676 |
| GOTERM_BP_DIRECT | GO:1903140~regulation of establishment of endothelial barrier | 2 | 3.448 | 0.026467676 |
| GOTERM_BP_DIRECT | GO:0001973~adenosine receptor signaling pathway | 2 | 3.448 | 0.026467676 |
| GOTERM_BP_DIRECT | GO:1902949~positive regulation of tau-protein kinase activity | 2 | 3.448 | 0.026467676 |
| GOTERM_BP_DIRECT | GO:0000902~cell morphogenesis | 3 | 5.172 | 0.02652229 |
| GOTERM_BP_DIRECT | GO:0030307~positive regulation of cell growth | 3 | 5.172 | 0.027688213 |
| GOTERM_BP_DIRECT | GO:0006805~xenobiotic metabolic process | 3 | 5.172 | 0.028278842 |
| GOTERM_BP_DIRECT | GO:0051247~positive regulation of protein metabolic process | 2 | 3.448 | 0.029365699 |
| GOTERM_BP_DIRECT | GO:0010863~positive regulation of phospholipase C activity | 2 | 3.448 | 0.029365699 |
| GOTERM_BP_DIRECT | GO:0002544~chronic inflammatory response | 2 | 3.448 | 0.029365699 |
| GOTERM_BP_DIRECT | GO:0045792~negative regulation of cell size | 2 | 3.448 | 0.029365699 |
| GOTERM_BP_DIRECT | GO:0006631~fatty acid metabolic process | 3 | 5.172 | 0.030080986 |
| GOTERM_BP_DIRECT | GO:0071492~cellular response to UV-A | 2 | 3.448 | 0.032255246 |
| GOTERM_BP_DIRECT | GO:0032308~positive regulation of prostaglandin secretion | 2 | 3.448 | 0.032255246 |
| GOTERM_BP_DIRECT | GO:1903799~negative regulation of production of miRNAs involved in gene silencing by miRNA | 2 | 3.448 | 0.032255246 |
| GOTERM_BP_DIRECT | GO:1900015~regulation of cytokine production involved in inflammatory response | 2 | 3.448 | 0.032255246 |
| GOTERM_BP_DIRECT | GO:0097011~cellular response to granulocyte macrophage colony-stimulating factor stimulus | 2 | 3.448 | 0.032255246 |
| GOTERM_BP_DIRECT | GO:0046330~positive regulation of JNK cascade | 3 | 5.172 | 0.033183382 |
| GOTERM_BP_DIRECT | GO:0006606~protein import into nucleus | 3 | 5.172 | 0.033183382 |
| GOTERM_BP_DIRECT | GO:0071346~cellular response to interferon-gamma | 3 | 5.172 | 0.035102642 |
| GOTERM_BP_DIRECT | GO:0051549~positive regulation of keratinocyte migration | 2 | 3.448 | 0.035136341 |
| GOTERM_BP_DIRECT | GO:0005979~regulation of glycogen biosynthetic process | 2 | 3.448 | 0.035136341 |
| GOTERM_BP_DIRECT | GO:1903800~positive regulation of production of miRNAs involved in gene silencing by miRNA | 2 | 3.448 | 0.035136341 |
| GOTERM_BP_DIRECT | GO:0050852~T cell receptor signaling pathway | 3 | 5.172 | 0.035751833 |
| GOTERM_BP_DIRECT | GO:0042632~cholesterol homeostasis | 3 | 5.172 | 0.03706418 |
| GOTERM_BP_DIRECT | GO:2000343~positive regulation of chemokine (C-X-C motif) ligand 2 production | 2 | 3.448 | 0.038009009 |
| GOTERM_BP_DIRECT | GO:0002675~positive regulation of acute inflammatory response | 2 | 3.448 | 0.038009009 |
| GOTERM_BP_DIRECT | GO:0097284~hepatocyte apoptotic process | 2 | 3.448 | 0.038009009 |
| GOTERM_BP_DIRECT | GO:1900272~negative regulation of long-term synaptic potentiation | 2 | 3.448 | 0.038009009 |
| GOTERM_BP_DIRECT | GO:0046902~regulation of mitochondrial membrane permeability | 2 | 3.448 | 0.038009009 |
| GOTERM_BP_DIRECT | GO:0045670~regulation of osteoclast differentiation | 2 | 3.448 | 0.038009009 |
| GOTERM_BP_DIRECT | GO:1902176~negative regulation of oxidative stress-induced intrinsic apoptotic signaling pathway | 2 | 3.448 | 0.040873274 |
| GOTERM_BP_DIRECT | GO:0055119~relaxation of cardiac muscle | 2 | 3.448 | 0.040873274 |
| GOTERM_BP_DIRECT | GO:0038183~bile acid signaling pathway | 2 | 3.448 | 0.040873274 |
| GOTERM_BP_DIRECT | GO:0060644~mammary gland epithelial cell differentiation | 2 | 3.448 | 0.040873274 |
| GOTERM_BP_DIRECT | GO:1904996~positive regulation of leukocyte adhesion to vascular endothelial cell | 2 | 3.448 | 0.040873274 |
| GOTERM_BP_DIRECT | GO:0006508~proteolysis | 5 | 8.621 | 0.041781695 |
| GOTERM_BP_DIRECT | GO:0090263~positive regulation of canonical Wnt signaling pathway | 3 | 5.172 | 0.041800621 |
| GOTERM_BP_DIRECT | GO:0008584~male gonad development | 3 | 5.172 | 0.04249502 |
| GOTERM_BP_DIRECT | GO:0009615~response to virus | 3 | 5.172 | 0.043193766 |
| GOTERM_BP_DIRECT | GO:0048266~behavioral response to pain | 2 | 3.448 | 0.04372916 |
| GOTERM_BP_DIRECT | GO:0010884~positive regulation of lipid storage | 2 | 3.448 | 0.04372916 |
| GOTERM_BP_DIRECT | GO:0032725~positive regulation of granulocyte macrophage colony-stimulating factor production | 2 | 3.448 | 0.04372916 |
| GOTERM_BP_DIRECT | GO:1900034~regulation of cellular response to heat | 2 | 3.448 | 0.04372916 |
| GOTERM_BP_DIRECT | GO:0010831~positive regulation of myotube differentiation | 2 | 3.448 | 0.04372916 |
| GOTERM_BP_DIRECT | GO:0045745~positive regulation of G-protein coupled receptor protein signaling pathway | 2 | 3.448 | 0.04372916 |
| GOTERM_BP_DIRECT | GO:0000165~MAPK cascade | 3 | 5.172 | 0.044604176 |
| GOTERM_BP_DIRECT | GO:0006974~cellular response to DNA damage stimulus | 4 | 6.897 | 0.045160156 |
| GOTERM_BP_DIRECT | GO:0050830~defense response to Gram-positive bacterium | 3 | 5.172 | 0.046031604 |
| GOTERM_BP_DIRECT | GO:0071383~cellular response to steroid hormone stimulus | 2 | 3.448 | 0.046576691 |
| GOTERM_BP_DIRECT | GO:0071639~positive regulation of monocyte chemotactic protein-1 production | 2 | 3.448 | 0.046576691 |
| GOTERM_BP_DIRECT | GO:0038084~vascular endothelial growth factor signaling pathway | 2 | 3.448 | 0.046576691 |
| GOTERM_BP_DIRECT | GO:0019395~fatty acid oxidation | 2 | 3.448 | 0.046576691 |
| GOTERM_BP_DIRECT | GO:0051403~stress-activated MAPK cascade | 2 | 3.448 | 0.046576691 |
| GOTERM_BP_DIRECT | GO:0008637~apoptotic mitochondrial changes | 2 | 3.448 | 0.046576691 |
| GOTERM_BP_DIRECT | GO:0006809~nitric oxide biosynthetic process | 2 | 3.448 | 0.046576691 |
| GOTERM_BP_DIRECT | GO:0034116~positive regulation of heterotypic cell-cell adhesion | 2 | 3.448 | 0.046576691 |
| GOTERM_BP_DIRECT | GO:0030278~regulation of ossification | 2 | 3.448 | 0.046576691 |
| GOTERM_BP_DIRECT | GO:0090336~positive regulation of brown fat cell differentiation | 2 | 3.448 | 0.046576691 |
| GOTERM_BP_DIRECT | GO:0032966~negative regulation of collagen biosynthetic process | 2 | 3.448 | 0.046576691 |
| GOTERM_BP_DIRECT | GO:0030224~monocyte differentiation | 2 | 3.448 | 0.049415891 |
| GOTERM_BP_DIRECT | GO:0055089~fatty acid homeostasis | 2 | 3.448 | 0.049415891 |
| GOTERM_BP_DIRECT | GO:0002407~dendritic cell chemotaxis | 2 | 3.448 | 0.049415891 |
| GOTERM_BP_DIRECT | GO:0071310~cellular response to organic substance | 2 | 3.448 | 0.049415891 |
| GOTERM_BP_DIRECT | GO:0050872~white fat cell differentiation | 2 | 3.448 | 0.049415891 |
| GOTERM_BP_DIRECT | GO:0045598~regulation of fat cell differentiation | 2 | 3.448 | 0.049415891 |
| GOTERM_BP_DIRECT | GO:0046889~positive regulation of lipid biosynthetic process | 2 | 3.448 | 0.052246784 |
| GOTERM_BP_DIRECT | GO:0045945~positive regulation of transcription from RNA polymerase III promoter | 2 | 3.448 | 0.052246784 |
| GOTERM_BP_DIRECT | GO:0003085~negative regulation of systemic arterial blood pressure | 2 | 3.448 | 0.052246784 |
| GOTERM_BP_DIRECT | GO:2000811~negative regulation of anoikis | 2 | 3.448 | 0.052246784 |
| GOTERM_BP_DIRECT | GO:0071391~cellular response to estrogen stimulus | 2 | 3.448 | 0.052246784 |
| GOTERM_BP_DIRECT | GO:0032481~positive regulation of type I interferon production | 2 | 3.448 | 0.055069394 |
| GOTERM_BP_DIRECT | GO:1902236~negative regulation of endoplasmic reticulum stress-induced intrinsic apoptotic signaling pathway | 2 | 3.448 | 0.055069394 |
| GOTERM_BP_DIRECT | GO:0010744~positive regulation of macrophage derived foam cell differentiation | 2 | 3.448 | 0.055069394 |
| GOTERM_BP_DIRECT | GO:0001516~prostaglandin biosynthetic process | 2 | 3.448 | 0.055069394 |
| GOTERM_BP_DIRECT | GO:0050728~negative regulation of inflammatory response | 3 | 5.172 | 0.055708054 |
| GOTERM_BP_DIRECT | GO:0030520~intracellular estrogen receptor signaling pathway | 2 | 3.448 | 0.057883745 |
| GOTERM_BP_DIRECT | GO:0031018~endocrine pancreas development | 2 | 3.448 | 0.057883745 |
| GOTERM_BP_DIRECT | GO:0031929~TOR signaling | 2 | 3.448 | 0.057883745 |
| GOTERM_BP_DIRECT | GO:0060716~labyrinthine layer blood vessel development | 2 | 3.448 | 0.057883745 |
| GOTERM_BP_DIRECT | GO:0038061~NIK/NF-kappaB signaling | 2 | 3.448 | 0.057883745 |
| GOTERM_BP_DIRECT | GO:0071404~cellular response to low-density lipoprotein particle stimulus | 2 | 3.448 | 0.057883745 |
| GOTERM_BP_DIRECT | GO:0033198~response to ATP | 2 | 3.448 | 0.057883745 |
| GOTERM_BP_DIRECT | GO:0010165~response to X-ray | 2 | 3.448 | 0.057883745 |
| GOTERM_BP_DIRECT | GO:0050901~leukocyte tethering or rolling | 2 | 3.448 | 0.057883745 |
| GOTERM_BP_DIRECT | GO:2000010~positive regulation of protein localization to cell surface | 2 | 3.448 | 0.057883745 |
| GOTERM_BP_DIRECT | GO:0045780~positive regulation of bone resorption | 2 | 3.448 | 0.057883745 |
| GOTERM_BP_DIRECT | GO:0018108~peptidyl-tyrosine phosphorylation | 3 | 5.172 | 0.058817936 |
| GOTERM_BP_DIRECT | GO:0090201~negative regulation of release of cytochrome c from mitochondria | 2 | 3.448 | 0.06068986 |
| GOTERM_BP_DIRECT | GO:0001659~temperature homeostasis | 2 | 3.448 | 0.06068986 |
| GOTERM_BP_DIRECT | GO:0036092~phosphatidylinositol-3-phosphate biosynthetic process | 2 | 3.448 | 0.06068986 |
| GOTERM_BP_DIRECT | GO:1902004~positive regulation of beta-amyloid formation | 2 | 3.448 | 0.06068986 |
| GOTERM_BP_DIRECT | GO:1902894~negative regulation of pri-miRNA transcription from RNA polymerase II promoter | 2 | 3.448 | 0.06068986 |
| GOTERM_BP_DIRECT | GO:0055013~cardiac muscle cell development | 2 | 3.448 | 0.06068986 |
| GOTERM_BP_DIRECT | GO:0035066~positive regulation of histone acetylation | 2 | 3.448 | 0.06068986 |
| GOTERM_BP_DIRECT | GO:0045672~positive regulation of osteoclast differentiation | 2 | 3.448 | 0.063487764 |
| GOTERM_BP_DIRECT | GO:0031641~regulation of myelination | 2 | 3.448 | 0.063487764 |
| GOTERM_BP_DIRECT | GO:0051090~regulation of sequence-specific DNA binding transcription factor activity | 2 | 3.448 | 0.066277479 |
| GOTERM_BP_DIRECT | GO:1900273~positive regulation of long-term synaptic potentiation | 2 | 3.448 | 0.066277479 |
| GOTERM_BP_DIRECT | GO:0090026~positive regulation of monocyte chemotaxis | 2 | 3.448 | 0.066277479 |
| GOTERM_BP_DIRECT | GO:0038095~Fc-epsilon receptor signaling pathway | 2 | 3.448 | 0.066277479 |
| GOTERM_BP_DIRECT | GO:0048714~positive regulation of oligodendrocyte differentiation | 2 | 3.448 | 0.066277479 |
| GOTERM_BP_DIRECT | GO:0060999~positive regulation of dendritic spine development | 2 | 3.448 | 0.069059029 |
| GOTERM_BP_DIRECT | GO:0010906~regulation of glucose metabolic process | 2 | 3.448 | 0.069059029 |
| GOTERM_BP_DIRECT | GO:0032740~positive regulation of interleukin-17 production | 2 | 3.448 | 0.069059029 |
| GOTERM_BP_DIRECT | GO:0035902~response to immobilization stress | 2 | 3.448 | 0.069059029 |
| GOTERM_BP_DIRECT | GO:0017144~drug metabolic process | 2 | 3.448 | 0.071832438 |
| GOTERM_BP_DIRECT | GO:0002639~positive regulation of immunoglobulin production | 2 | 3.448 | 0.071832438 |
| GOTERM_BP_DIRECT | GO:1905564~positive regulation of vascular endothelial cell proliferation | 2 | 3.448 | 0.071832438 |
| GOTERM_BP_DIRECT | GO:0008283~cell proliferation | 3 | 5.172 | 0.073518927 |
| GOTERM_BP_DIRECT | GO:0002092~positive regulation of receptor internalization | 2 | 3.448 | 0.074597729 |
| GOTERM_BP_DIRECT | GO:0071385~cellular response to glucocorticoid stimulus | 2 | 3.448 | 0.074597729 |
| GOTERM_BP_DIRECT | GO:0002862~negative regulation of inflammatory response to antigenic stimulus | 2 | 3.448 | 0.074597729 |
| GOTERM_BP_DIRECT | GO:0048010~vascular endothelial growth factor receptor signaling pathway | 2 | 3.448 | 0.077354926 |
| GOTERM_BP_DIRECT | GO:0010875~positive regulation of cholesterol efflux | 2 | 3.448 | 0.077354926 |
| GOTERM_BP_DIRECT | GO:0051968~positive regulation of synaptic transmission, glutamatergic | 2 | 3.448 | 0.077354926 |
| GOTERM_BP_DIRECT | GO:0007569~cell aging | 2 | 3.448 | 0.082845129 |
| GOTERM_BP_DIRECT | GO:0071280~cellular response to copper ion | 2 | 3.448 | 0.082845129 |
| GOTERM_BP_DIRECT | GO:0010592~positive regulation of lamellipodium assembly | 2 | 3.448 | 0.082845129 |
| GOTERM_BP_DIRECT | GO:0001774~microglial cell activation | 2 | 3.448 | 0.082845129 |
| GOTERM_BP_DIRECT | GO:0043200~response to amino acid | 2 | 3.448 | 0.085578181 |
| GOTERM_BP_DIRECT | GO:0060612~adipose tissue development | 2 | 3.448 | 0.085578181 |
| GOTERM_BP_DIRECT | GO:0007159~leukocyte cell-cell adhesion | 2 | 3.448 | 0.085578181 |
| GOTERM_BP_DIRECT | GO:0010800~positive regulation of peptidyl-threonine phosphorylation | 2 | 3.448 | 0.085578181 |
| GOTERM_BP_DIRECT | GO:0045840~positive regulation of mitotic nuclear division | 2 | 3.448 | 0.085578181 |
| GOTERM_BP_DIRECT | GO:0035633~maintenance of permeability of blood-brain barrier | 2 | 3.448 | 0.088303232 |
| GOTERM_BP_DIRECT | GO:0030316~osteoclast differentiation | 2 | 3.448 | 0.091020304 |
| GOTERM_BP_DIRECT | GO:1900745~positive regulation of p38MAPK cascade | 2 | 3.448 | 0.091020304 |
| GOTERM_BP_DIRECT | GO:0045087~innate immune response | 5 | 8.621 | 0.092873812 |
| GOTERM_BP_DIRECT | GO:0043388~positive regulation of DNA binding | 2 | 3.448 | 0.09372942 |
| GOTERM_BP_DIRECT | GO:0045737~positive regulation of cyclin-dependent protein serine/threonine kinase activity | 2 | 3.448 | 0.09372942 |
| GOTERM_BP_DIRECT | GO:0045648~positive regulation of erythrocyte differentiation | 2 | 3.448 | 0.09372942 |
| GOTERM_BP_DIRECT | GO:0045765~regulation of angiogenesis | 2 | 3.448 | 0.09372942 |
| GOTERM_BP_DIRECT | GO:0045739~positive regulation of DNA repair | 2 | 3.448 | 0.09372942 |
| GOTERM_BP_DIRECT | GO:0045892~negative regulation of transcription, DNA-templated | 5 | 8.621 | 0.093769142 |
| GOTERM_BP_DIRECT | GO:0021510~spinal cord development | 2 | 3.448 | 0.096430602 |
| GOTERM_BP_DIRECT | GO:0002230~positive regulation of defense response to virus by host | 2 | 3.448 | 0.096430602 |
| GOTERM_BP_DIRECT | GO:0051973~positive regulation of telomerase activity | 2 | 3.448 | 0.096430602 |
| GOTERM_BP_DIRECT | GO:0034612~response to tumor necrosis factor | 2 | 3.448 | 0.099123875 |
| GOTERM_BP_DIRECT | GO:0045930~negative regulation of mitotic cell cycle | 2 | 3.448 | 0.099123875 |
| GOTERM_CC_DIRECT | GO:0032991~macromolecular complex | 15 | 25.862 | 5.32E-09 |
| GOTERM_CC_DIRECT | GO:0005737~cytoplasm | 37 | 63.793 | 8.05E-09 |
| GOTERM_CC_DIRECT | GO:0009986~cell surface | 11 | 18.966 | 1.00E-05 |
| GOTERM_CC_DIRECT | GO:0005886~plasma membrane | 30 | 51.724 | 1.80E-05 |
| GOTERM_CC_DIRECT | GO:0005768~endosome | 8 | 13.793 | 2.65E-05 |
| GOTERM_CC_DIRECT | GO:0005829~cytosol | 31 | 53.448 | 2.86E-05 |
| GOTERM_CC_DIRECT | GO:0045202~synapse | 8 | 13.793 | 3.78E-04 |
| GOTERM_CC_DIRECT | GO:0005576~extracellular region | 16 | 27.586 | 6.03E-04 |
| GOTERM_CC_DIRECT | GO:0045121~membrane raft | 6 | 10.345 | 7.10E-04 |
| GOTERM_CC_DIRECT | GO:0005769~early endosome | 6 | 10.345 | 0.001271 |
| GOTERM_CC_DIRECT | GO:0005654~nucleoplasm | 22 | 37.931 | 0.001293 |
| GOTERM_CC_DIRECT | GO:0005615~extracellular space | 14 | 24.138 | 0.002147 |
| GOTERM_CC_DIRECT | GO:0005887~integral component of plasma membrane | 12 | 20.690 | 0.002162 |
| GOTERM_CC_DIRECT | GO:0000785~chromatin | 10 | 17.241 | 0.002284 |
| GOTERM_CC_DIRECT | GO:0048471~perinuclear region of cytoplasm | 8 | 13.793 | 0.005177 |
| GOTERM_CC_DIRECT | GO:1904813~ficolin-1-rich granule lumen | 4 | 6.897 | 0.005216 |
| GOTERM_CC_DIRECT | GO:0043025~neuronal cell body | 6 | 10.345 | 0.005275 |
| GOTERM_CC_DIRECT | GO:0005739~mitochondrion | 11 | 18.966 | 0.00571 |
| GOTERM_CC_DIRECT | GO:0005788~endoplasmic reticulum lumen | 5 | 8.621 | 0.011058 |
| GOTERM_CC_DIRECT | GO:0005944~phosphatidylinositol 3-kinase complex, class IB | 2 | 3.448 | 0.011261 |
| GOTERM_CC_DIRECT | GO:0005634~nucleus | 26 | 44.828 | 0.011877 |
| GOTERM_CC_DIRECT | GO:0005943~phosphatidylinositol 3-kinase complex, class IA | 2 | 3.448 | 0.016844 |
| GOTERM_CC_DIRECT | GO:0016020~membrane | 14 | 24.138 | 0.021395 |
| GOTERM_CC_DIRECT | GO:0043235~receptor complex | 4 | 6.897 | 0.023224 |
| GOTERM_CC_DIRECT | GO:0005641~nuclear envelope lumen | 2 | 3.448 | 0.027919 |
| GOTERM_CC_DIRECT | GO:0005925~focal adhesion | 5 | 8.621 | 0.031311 |
| GOTERM_CC_DIRECT | GO:0009897~external side of plasma membrane | 5 | 8.621 | 0.037217 |
| GOTERM_CC_DIRECT | GO:0090575~RNA polymerase II transcription factor complex | 3 | 5.172 | 0.040685 |
| GOTERM_CC_DIRECT | GO:0034774~secretory granule lumen | 3 | 5.172 | 0.041994 |
| GOTERM_CC_DIRECT | GO:0055037~recycling endosome | 3 | 5.172 | 0.053742 |
| GOTERM_CC_DIRECT | GO:0005942~phosphatidylinositol 3-kinase complex | 2 | 3.448 | 0.060412 |
| GOTERM_CC_DIRECT | GO:0005815~microtubule organizing center | 3 | 5.172 | 0.072025 |
| GOTERM_CC_DIRECT | GO:0001891~phagocytic cup | 2 | 3.448 | 0.076256 |
| GOTERM_CC_DIRECT | GO:0030424~axon | 4 | 6.897 | 0.077506 |
| GOTERM_CC_DIRECT | GO:0016324~apical plasma membrane | 4 | 6.897 | 0.086436 |
| GOTERM_CC_DIRECT | GO:0005783~endoplasmic reticulum | 7 | 12.069 | 0.092925 |
| GOTERM_MF_DIRECT | GO:0019899~enzyme binding | 13 | 22.414 | 1.32E-09 |
| GOTERM_MF_DIRECT | GO:0004879~RNA polymerase II transcription factor activity, ligand-activated sequence-specific DNA binding | 7 | 12.069 | 1.38E-08 |
| GOTERM_MF_DIRECT | GO:0042802~identical protein binding | 20 | 34.483 | 2.87E-07 |
| GOTERM_MF_DIRECT | GO:0030235~nitric-oxide synthase regulator activity | 4 | 6.897 | 2.20E-06 |
| GOTERM_MF_DIRECT | GO:0004674~protein serine/threonine kinase activity | 10 | 17.241 | 2.51E-06 |
| GOTERM_MF_DIRECT | GO:0004712~protein serine/threonine/tyrosine kinase activity | 10 | 17.241 | 5.44E-06 |
| GOTERM_MF_DIRECT | GO:0019903~protein phosphatase binding | 6 | 10.345 | 8.69E-06 |
| GOTERM_MF_DIRECT | GO:0005515~protein binding | 53 | 91.379 | 1.47E-05 |
| GOTERM_MF_DIRECT | GO:0005524~ATP binding | 16 | 27.586 | 3.91E-05 |
| GOTERM_MF_DIRECT | GO:0019901~protein kinase binding | 9 | 15.517 | 1.24E-04 |
| GOTERM_MF_DIRECT | GO:0005178~integrin binding | 6 | 10.345 | 1.28E-04 |
| GOTERM_MF_DIRECT | GO:0004672~protein kinase activity | 8 | 13.793 | 1.41E-04 |
| GOTERM_MF_DIRECT | GO:0003700~transcription factor activity, sequence-specific DNA binding | 9 | 15.517 | 2.51E-04 |
| GOTERM_MF_DIRECT | GO:0042803~protein homodimerization activity | 10 | 17.241 | 2.53E-04 |
| GOTERM_MF_DIRECT | GO:0005125~cytokine activity | 6 | 10.345 | 2.76E-04 |
| GOTERM_MF_DIRECT | GO:0004707~MAP kinase activity | 3 | 5.172 | 9.28E-04 |
| GOTERM_MF_DIRECT | GO:0000978~RNA polymerase II core promoter proximal region sequence-specific DNA binding | 11 | 18.966 | 0.003119 |
| GOTERM_MF_DIRECT | GO:0043565~sequence-specific DNA binding | 6 | 10.345 | 0.003151 |
| GOTERM_MF_DIRECT | GO:0008134~transcription factor binding | 5 | 8.621 | 0.003599 |
| GOTERM_MF_DIRECT | GO:0005496~steroid binding | 3 | 5.172 | 0.003734 |
| GOTERM_MF_DIRECT | GO:0046875~ephrin receptor binding | 3 | 5.172 | 0.003734 |
| GOTERM_MF_DIRECT | GO:0008270~zinc ion binding | 9 | 15.517 | 0.0044 |
| GOTERM_MF_DIRECT | GO:0003677~DNA binding | 11 | 18.966 | 0.004821 |
| GOTERM_MF_DIRECT | GO:0016301~kinase activity | 5 | 8.621 | 0.004913 |
| GOTERM_MF_DIRECT | GO:0042056~chemoattractant activity | 3 | 5.172 | 0.005345 |
| GOTERM_MF_DIRECT | GO:0000976~transcription regulatory region sequence-specific DNA binding | 5 | 8.621 | 0.00572 |
| GOTERM_MF_DIRECT | GO:0005102~receptor binding | 6 | 10.345 | 0.007099 |
| GOTERM_MF_DIRECT | GO:0001223~transcription coactivator binding | 3 | 5.172 | 0.007902 |
| GOTERM_MF_DIRECT | GO:0090722~receptor-receptor interaction | 2 | 3.448 | 0.012096 |
| GOTERM_MF_DIRECT | GO:0003682~chromatin binding | 6 | 10.345 | 0.013311 |
| GOTERM_MF_DIRECT | GO:0031625~ubiquitin protein ligase binding | 5 | 8.621 | 0.013613 |
| GOTERM_MF_DIRECT | GO:0001228~transcriptional activator activity, RNA polymerase II transcription regulatory region sequence-specific binding | 6 | 10.345 | 0.014004 |
| GOTERM_MF_DIRECT | GO:0008083~growth factor activity | 4 | 6.897 | 0.014031 |
| GOTERM_MF_DIRECT | GO:0035004~phosphatidylinositol 3-kinase activity | 2 | 3.448 | 0.015097 |
| GOTERM_MF_DIRECT | GO:0001609~G-protein coupled adenosine receptor activity | 2 | 3.448 | 0.015097 |
| GOTERM_MF_DIRECT | GO:0008201~heparin binding | 4 | 6.897 | 0.01542 |
| GOTERM_MF_DIRECT | GO:0042277~peptide binding | 3 | 5.172 | 0.017665 |
| GOTERM_MF_DIRECT | GO:0001664~G-protein coupled receptor binding | 3 | 5.172 | 0.019702 |
| GOTERM_MF_DIRECT | GO:0035005~1-phosphatidylinositol-4-phosphate 3-kinase activity | 2 | 3.448 | 0.021074 |
| GOTERM_MF_DIRECT | GO:0046934~phosphatidylinositol-4,5-bisphosphate 3-kinase activity | 2 | 3.448 | 0.021074 |
| GOTERM_MF_DIRECT | GO:0031730~CCR5 chemokine receptor binding | 2 | 3.448 | 0.024048 |
| GOTERM_MF_DIRECT | GO:0052812~phosphatidylinositol-3,4-bisphosphate 5-kinase activity | 2 | 3.448 | 0.027014 |
| GOTERM_MF_DIRECT | GO:0004175~endopeptidase activity | 3 | 5.172 | 0.028154 |
| GOTERM_MF_DIRECT | GO:0016303~1-phosphatidylinositol-3-kinase activity | 2 | 3.448 | 0.029971 |
| GOTERM_MF_DIRECT | GO:0097199~cysteine-type endopeptidase activity involved in apoptotic signaling pathway | 2 | 3.448 | 0.029971 |
| GOTERM_MF_DIRECT | GO:0051117~ATPase binding | 3 | 5.172 | 0.030613 |
| GOTERM_MF_DIRECT | GO:0052742~phosphatidylinositol kinase activity | 2 | 3.448 | 0.035858 |
| GOTERM_MF_DIRECT | GO:0019900~kinase binding | 3 | 5.172 | 0.037118 |
| GOTERM_MF_DIRECT | GO:0000981~RNA polymerase II transcription factor activity, sequence-specific DNA binding | 9 | 15.517 | 0.038451 |
| GOTERM_MF_DIRECT | GO:0008233~peptidase activity | 3 | 5.172 | 0.039165 |
| GOTERM_MF_DIRECT | GO:0002020~protease binding | 3 | 5.172 | 0.042669 |
| GOTERM_MF_DIRECT | GO:0004713~protein tyrosine kinase activity | 3 | 5.172 | 0.044102 |
| GOTERM_MF_DIRECT | GO:0008559~xenobiotic-transporting ATPase activity | 2 | 3.448 | 0.044624 |
| GOTERM_MF_DIRECT | GO:0019904~protein domain specific binding | 4 | 6.897 | 0.045352 |
| GOTERM_MF_DIRECT | GO:0003690~double-stranded DNA binding | 3 | 5.172 | 0.047763 |
| GOTERM_MF_DIRECT | GO:0004714~transmembrane receptor protein tyrosine kinase activity | 3 | 5.172 | 0.048509 |
| GOTERM_MF_DIRECT | GO:0046965~retinoid X receptor binding | 2 | 3.448 | 0.053311 |
| GOTERM_MF_DIRECT | GO:0004708~MAP kinase kinase activity | 2 | 3.448 | 0.053311 |
| GOTERM_MF_DIRECT | GO:0004950~chemokine receptor activity | 2 | 3.448 | 0.056189 |
| GOTERM_MF_DIRECT | GO:0034185~apolipoprotein binding | 2 | 3.448 | 0.056189 |
| GOTERM_MF_DIRECT | GO:0043395~heparan sulfate proteoglycan binding | 2 | 3.448 | 0.056189 |
| GOTERM_MF_DIRECT | GO:0016493~C-C chemokine receptor activity | 2 | 3.448 | 0.067617 |
| GOTERM_MF_DIRECT | GO:0005158~insulin receptor binding | 2 | 3.448 | 0.070453 |
| GOTERM_MF_DIRECT | GO:0019957~C-C chemokine binding | 2 | 3.448 | 0.070453 |
| GOTERM_MF_DIRECT | GO:0020037~heme binding | 3 | 5.172 | 0.076184 |
| GOTERM_MF_DIRECT | GO:0001227~transcriptional repressor activity, RNA polymerase II transcription regulatory region sequence-specific binding | 4 | 6.897 | 0.078817 |
| GOTERM_MF_DIRECT | GO:0046872~metal ion binding | 13 | 22.414 | 0.094936 |

**Supplementary Table S4. KEGG enrichment analysis results**

| **Category** | **Term** | **Count** | **%** | **P Value** |
| --- | --- | --- | --- | --- |
| KEGG_PATHWAY | hsa05167:Kaposi sarcoma-associated herpesvirus infection | 18 | 31.034 | 2.76E-15 |
| KEGG_PATHWAY | hsa05200:Pathways in cancer | 25 | 43.103 | 4.65E-15 |
| KEGG_PATHWAY | hsa04933:AGE-RAGE signaling pathway in diabetic complications | 14 | 24.138 | 4.76E-14 |
| KEGG_PATHWAY | hsa04630:JAK-STAT signaling pathway | 14 | 24.138 | 8.03E-14 |
| KEGG_PATHWAY | hsa04064:NF-kappa B signaling pathway | 17 | 29.310 | 2.78E-13 |
| KEGG_PATHWAY | hsa05163:Human cytomegalovirus infection | 17 | 29.310 | 5.65E-13 |
| KEGG_PATHWAY | hsa04668:TNF signaling pathway | 13 | 22.414 | 5.33E-12 |
| KEGG_PATHWAY | hsa05212:Pancreatic cancer | 11 | 18.966 | 4.70E-11 |
| KEGG_PATHWAY | hsa01521:EGFR tyrosine kinase inhibitor resistance | 11 | 18.966 | 6.99E-11 |
| KEGG_PATHWAY | hsa04659:Th17 cell differentiation | 12 | 20.690 | 7.93E-11 |
| KEGG_PATHWAY | hsa04151:PI3K-Akt signaling pathway | 12 | 20.690 | 1.18E-10 |
| KEGG_PATHWAY | hsa04657:IL-17 signaling pathway | 11 | 18.966 | 4.07E-10 |
| KEGG_PATHWAY | hsa05161:Hepatitis B | 13 | 22.414 | 4.26E-10 |
| KEGG_PATHWAY | hsa04380:Osteoclast differentiation | 12 | 20.690 | 5.07E-10 |
| KEGG_PATHWAY | hsa05207:Chemical carcinogenesis - receptor activation | 14 | 24.138 | 7.57E-10 |
| KEGG_PATHWAY | hsa05210:Colorectal cancer | 11 | 18.966 | 9.22E-10 |
| KEGG_PATHWAY | hsa04210:Apoptosis | 12 | 20.690 | 1.23E-09 |
| KEGG_PATHWAY | hsa05133:Pertussis | 10 | 17.241 | 1.26E-09 |
| KEGG_PATHWAY | hsa05152:Tuberculosis | 13 | 22.414 | 1.44E-09 |
| KEGG_PATHWAY | hsa05171:Coronavirus disease - COVID-19 | 14 | 24.138 | 2.30E-09 |
| KEGG_PATHWAY | hsa05145:Toxoplasmosis | 16 | 27.586 | 5.14E-09 |
| KEGG_PATHWAY | hsa05169:Epstein-Barr virus infection | 13 | 22.414 | 5.39E-09 |
| KEGG_PATHWAY | hsa05205:Proteoglycans in cancer | 13 | 22.414 | 6.38E-09 |
| KEGG_PATHWAY | hsa04068:FoxO signaling pathway | 11 | 18.966 | 1.08E-08 |
| KEGG_PATHWAY | hsa05215:Prostate cancer | 10 | 17.241 | 1.13E-08 |
| KEGG_PATHWAY | hsa01522:Endocrine resistance | 10 | 17.241 | 1.24E-08 |
| KEGG_PATHWAY | hsa05135:Yersinia infection | 11 | 18.966 | 1.67E-08 |
| KEGG_PATHWAY | hsa05418:Fluid shear stress and atherosclerosis | 11 | 18.966 | 1.92E-08 |
| KEGG_PATHWAY | hsa04066:HIF-1 signaling pathway | 10 | 17.241 | 3.19E-08 |
| KEGG_PATHWAY | hsa05132:Salmonella infection | 13 | 22.414 | 5.63E-08 |
| KEGG_PATHWAY | hsa04071:Sphingolipid signaling pathway | 10 | 17.241 | 6.87E-08 |
| KEGG_PATHWAY | hsa05235:PD-L1 expression and PD-1 checkpoint pathway in cancer | 9 | 15.517 | 1.02E-07 |
| KEGG_PATHWAY | hsa05010:Alzheimer disease | 15 | 25.862 | 1.23E-07 |
| KEGG_PATHWAY | hsa05164:Influenza A | 11 | 18.966 | 1.38E-07 |
| KEGG_PATHWAY | hsa04926:Relaxin signaling pathway | 10 | 17.241 | 1.38E-07 |
| KEGG_PATHWAY | hsa05166:Human T-cell leukemia virus 1 infection | 12 | 20.690 | 1.65E-07 |
| KEGG_PATHWAY | hsa04621:NOD-like receptor signaling pathway | 11 | 18.966 | 2.75E-07 |
| KEGG_PATHWAY | hsa04936:Alcoholic liver disease | 10 | 17.241 | 3.16E-07 |
| KEGG_PATHWAY | hsa04917:Prolactin signaling pathway | 8 | 13.793 | 3.29E-07 |
| KEGG_PATHWAY | hsa04620:Toll-like receptor signaling pathway | 9 | 15.517 | 3.46E-07 |
| KEGG_PATHWAY | hsa04931:Insulin resistance | 9 | 15.517 | 4.63E-07 |
| KEGG_PATHWAY | hsa05131:Shigellosis | 12 | 20.690 | 4.84E-07 |
| KEGG_PATHWAY | hsa05140:Leishmaniasis | 8 | 13.793 | 6.36E-07 |
| KEGG_PATHWAY | hsa04932:Non-alcoholic fatty liver disease | 10 | 17.241 | 6.66E-07 |
| KEGG_PATHWAY | hsa05160:Hepatitis C | 10 | 17.241 | 7.42E-07 |
| KEGG_PATHWAY | hsa05134:Legionellosis | 7 | 12.069 | 1.79E-06 |
| KEGG_PATHWAY | hsa05222:Small cell lung cancer | 8 | 13.793 | 2.15E-06 |
| KEGG_PATHWAY | hsa04010:MAPK signaling pathway | 12 | 20.690 | 2.70E-06 |
| KEGG_PATHWAY | hsa05321:Inflammatory bowel disease | 7 | 12.069 | 3.92E-06 |
| KEGG_PATHWAY | hsa05206:MicroRNAs in cancer | 12 | 20.690 | 4.51E-06 |
| KEGG_PATHWAY | hsa05221:Acute myeloid leukemia | 7 | 12.069 | 4.68E-06 |
| KEGG_PATHWAY | hsa04660:T cell receptor signaling pathway | 8 | 13.793 | 4.90E-06 |
| KEGG_PATHWAY | hsa05417:Lipid and atherosclerosis | 8 | 13.793 | 4.90E-06 |
| KEGG_PATHWAY | hsa04664:Fc epsilon RI signaling pathway | 7 | 12.069 | 5.11E-06 |
| KEGG_PATHWAY | hsa05203:Viral carcinogenesis | 10 | 17.241 | 6.53E-06 |
| KEGG_PATHWAY | hsa04218:Cellular senescence | 9 | 15.517 | 7.48E-06 |
| KEGG_PATHWAY | hsa05165:Human papillomavirus infection | 12 | 20.690 | 8.46E-06 |
| KEGG_PATHWAY | hsa05170:Human immunodeficiency virus 1 infection | 10 | 17.241 | 8.93E-06 |
| KEGG_PATHWAY | hsa04625:C-type lectin receptor signaling pathway | 9 | 15.517 | 9.88E-06 |
| KEGG_PATHWAY | hsa05208:Chemical carcinogenesis - reactive oxygen species | 10 | 17.241 | 1.34E-05 |
| KEGG_PATHWAY | hsa05142:Chagas disease | 7 | 12.069 | 2.00E-05 |
| KEGG_PATHWAY | hsa05162:Measles | 8 | 13.793 | 2.86E-05 |
| KEGG_PATHWAY | hsa04370:VEGF signaling pathway | 6 | 10.345 | 4.05E-05 |
| KEGG_PATHWAY | hsa05130:Pathogenic Escherichia coli infection | 9 | 15.517 | 4.08E-05 |
| KEGG_PATHWAY | hsa05224:Breast cancer | 8 | 13.793 | 4.72E-05 |
| KEGG_PATHWAY | hsa05022:Pathways of neurodegeneration - multiple diseases | 13 | 22.414 | 5.00E-05 |
| KEGG_PATHWAY | hsa05226:Gastric cancer | 8 | 13.793 | 5.14E-05 |
| KEGG_PATHWAY | hsa05146:Amoebiasis | 7 | 12.069 | 5.29E-05 |
| KEGG_PATHWAY | hsa04914:Progesterone-mediated oocyte maturation | 7 | 12.069 | 5.29E-05 |
| KEGG_PATHWAY | hsa04920:Adipocytokine signaling pathway | 6 | 10.345 | 8.65E-05 |
| KEGG_PATHWAY | hsa04935:Growth hormone synthesis, secretion and action | 7 | 12.069 | 1.25E-04 |
| KEGG_PATHWAY | hsa04014:Ras signaling pathway | 9 | 15.517 | 1.29E-04 |
| KEGG_PATHWAY | hsa04930:Type II diabetes mellitus | 5 | 8.621 | 2.29E-04 |
| KEGG_PATHWAY | hsa04012:ErbB signaling pathway | 6 | 10.345 | 2.33E-04 |
| KEGG_PATHWAY | hsa04613:Neutrophil extracellular trap formation | 8 | 13.793 | 2.36E-04 |
| KEGG_PATHWAY | hsa04062:Chemokine signaling pathway | 8 | 13.793 | 2.52E-04 |
| KEGG_PATHWAY | hsa04910:Insulin signaling pathway | 7 | 12.069 | 2.71E-04 |
| KEGG_PATHWAY | hsa04915:Estrogen signaling pathway | 7 | 12.069 | 2.81E-04 |
| KEGG_PATHWAY | hsa04211:Longevity regulating pathway | 6 | 10.345 | 2.89E-04 |
| KEGG_PATHWAY | hsa04658:Th1 and Th2 cell differentiation | 6 | 10.345 | 3.37E-04 |
| KEGG_PATHWAY | hsa04550:Signaling pathways regulating pluripotency of stem cells | 7 | 12.069 | 3.41E-04 |
| KEGG_PATHWAY | hsa04072:Phospholipase D signaling pathway | 7 | 12.069 | 4.10E-04 |
| KEGG_PATHWAY | hsa05231:Choline metabolism in cancer | 6 | 10.345 | 4.52E-04 |
| KEGG_PATHWAY | hsa04217:Necroptosis | 7 | 12.069 | 6.01E-04 |
| KEGG_PATHWAY | hsa01523:Antifolate resistance | 4 | 6.897 | 9.86E-04 |
| KEGG_PATHWAY | hsa04722:Neurotrophin signaling pathway | 6 | 10.345 | 0.001095 |
| KEGG_PATHWAY | hsa04152:AMPK signaling pathway | 6 | 10.345 | 0.001137 |
| KEGG_PATHWAY | hsa05120:Epithelial cell signaling in Helicobacter pylori infection | 5 | 8.621 | 0.001146 |
| KEGG_PATHWAY | hsa05230:Central carbon metabolism in cancer | 5 | 8.621 | 0.001146 |
| KEGG_PATHWAY | hsa05218:Melanoma | 5 | 8.621 | 0.001273 |
| KEGG_PATHWAY | hsa05223:Non-small cell lung cancer | 5 | 8.621 | 0.001273 |
| KEGG_PATHWAY | hsa04611:Platelet activation | 6 | 10.345 | 0.001316 |
| KEGG_PATHWAY | hsa05168:Herpes simplex virus 1 infection | 11 | 18.966 | 0.001334 |
| KEGG_PATHWAY | hsa01524:Platinum drug resistance | 5 | 8.621 | 0.001341 |
| KEGG_PATHWAY | hsa05214:Glioma | 5 | 8.621 | 0.001483 |
| KEGG_PATHWAY | hsa05220:Chronic myeloid leukemia | 5 | 8.621 | 0.001557 |
| KEGG_PATHWAY | hsa05143:African trypanosomiasis | 4 | 6.897 | 0.001827 |
| KEGG_PATHWAY | hsa05020:Prion disease | 8 | 13.793 | 0.002026 |
| KEGG_PATHWAY | hsa04662:B cell receptor signaling pathway | 5 | 8.621 | 0.002062 |
| KEGG_PATHWAY | hsa05415:Diabetic cardiomyopathy | 7 | 12.069 | 0.002127 |
| KEGG_PATHWAY | hsa04140:Autophagy - animal | 6 | 10.345 | 0.002321 |
| KEGG_PATHWAY | hsa05219:Bladder cancer | 4 | 6.897 | 0.00246 |
| KEGG_PATHWAY | hsa04015:Rap1 signaling pathway | 7 | 12.069 | 0.002522 |
| KEGG_PATHWAY | hsa05332:Graft-versus-host disease | 4 | 6.897 | 0.002637 |
| KEGG_PATHWAY | hsa04024:cAMP signaling pathway | 7 | 12.069 | 0.003252 |
| KEGG_PATHWAY | hsa04061:Viral protein interaction with cytokine and cytokine receptor | 5 | 8.621 | 0.004234 |
| KEGG_PATHWAY | hsa05144:Malaria | 4 | 6.897 | 0.004337 |
| KEGG_PATHWAY | hsa04022:cGMP-PKG signaling pathway | 6 | 10.345 | 0.004805 |
| KEGG_PATHWAY | hsa05225:Hepatocellular carcinoma | 6 | 10.345 | 0.004928 |
| KEGG_PATHWAY | hsa04923:Regulation of lipolysis in adipocytes | 4 | 6.897 | 0.005965 |
| KEGG_PATHWAY | hsa05213:Endometrial cancer | 4 | 6.897 | 0.006579 |
| KEGG_PATHWAY | hsa04726:Serotonergic synapse | 5 | 8.621 | 0.006939 |
| KEGG_PATHWAY | hsa04213:Longevity regulating pathway - multiple species | 4 | 6.897 | 0.007915 |
| KEGG_PATHWAY | hsa04623:Cytosolic DNA-sensing pathway | 4 | 6.897 | 0.008272 |
| KEGG_PATHWAY | hsa04919:Thyroid hormone signaling pathway | 5 | 8.621 | 0.008282 |
| KEGG_PATHWAY | hsa04650:Natural killer cell mediated cytotoxicity | 5 | 8.621 | 0.009524 |
| KEGG_PATHWAY | hsa04510:Focal adhesion | 6 | 10.345 | 0.010354 |
| KEGG_PATHWAY | hsa05211:Renal cell carcinoma | 4 | 6.897 | 0.010614 |
| KEGG_PATHWAY | hsa04622:RIG-I-like receptor signaling pathway | 4 | 6.897 | 0.011038 |
| KEGG_PATHWAY | hsa04371:Apelin signaling pathway | 5 | 8.621 | 0.013304 |
| KEGG_PATHWAY | hsa04215:Apoptosis - multiple species | 3 | 5.172 | 0.018858 |
| KEGG_PATHWAY | hsa04150:mTOR signaling pathway | 5 | 8.621 | 0.019533 |
| KEGG_PATHWAY | hsa04912:GnRH signaling pathway | 4 | 6.897 | 0.023527 |
| KEGG_PATHWAY | hsa05323:Rheumatoid arthritis | 4 | 6.897 | 0.023527 |
| KEGG_PATHWAY | hsa04666:Fc gamma R-mediated phagocytosis | 4 | 6.897 | 0.026243 |
| KEGG_PATHWAY | hsa04750:Inflammatory mediator regulation of TRP channels | 4 | 6.897 | 0.026948 |
| KEGG_PATHWAY | hsa04640:Hematopoietic cell lineage | 4 | 6.897 | 0.027662 |
| KEGG_PATHWAY | hsa05014:Amyotrophic lateral sclerosis | 7 | 12.069 | 0.032457 |
| KEGG_PATHWAY | hsa04940:Type I diabetes mellitus | 3 | 5.172 | 0.032782 |
| KEGG_PATHWAY | hsa05202:Transcriptional misregulation in cancer | 5 | 8.621 | 0.038578 |
| KEGG_PATHWAY | hsa04725:Cholinergic synapse | 4 | 6.897 | 0.03872 |
| KEGG_PATHWAY | hsa04670:Leukocyte transendothelial migration | 4 | 6.897 | 0.039585 |
| KEGG_PATHWAY | hsa04060:Cytokine-cytokine receptor interaction | 6 | 10.345 | 0.044994 |
| KEGG_PATHWAY | hsa04114:Oocyte meiosis | 4 | 6.897 | 0.055766 |
| KEGG_PATHWAY | hsa04929:GnRH secretion | 3 | 5.172 | 0.067056 |
| KEGG_PATHWAY | hsa05017:Spinocerebellar ataxia | 4 | 6.897 | 0.068814 |
| KEGG_PATHWAY | hsa04723:Retrograde endocannabinoid signaling | 4 | 6.897 | 0.074628 |
| KEGG_PATHWAY | hsa04261:Adrenergic signaling in cardiomyocytes | 4 | 6.897 | 0.077013 |
| KEGG_PATHWAY | hsa04520:Adherens junction | 3 | 5.172 | 0.080295 |
| KEGG_PATHWAY | hsa04921:Oxytocin signaling pathway | 4 | 6.897 | 0.081885 |
| KEGG_PATHWAY | hsa04115:p53 signaling pathway | 3 | 5.172 | 0.084217 |
| KEGG_PATHWAY | hsa04080:Neuroactive ligand-receptor interaction | 6 | 10.345 | 0.090699 |
